# Medium‐Entropy Alloy/Oxide Nano Composite for High‐Performing High‐Temperature CO_2_ Electrolysis with Remarkable Carbon Deposition Resistance

**DOI:** 10.1002/advs.202508800

**Published:** 2025-07-29

**Authors:** Jun Tong, Haewon Seo, Yunseo Choi, Ji‐eun Won, Jinhong Park, Keun Hwa Chae, Jongsup Hong, Hye Jung Chang, Baowen Zhou, Rongchang Cao, Na Ni, Kyung Joong Yoon, Lei Zhu, Zhen Huang

**Affiliations:** ^1^ Key Laboratory for Power Machinery and Engineering of Ministry of Education Shanghai Jiao Tong University Shanghai 200240 China; ^2^ Center for Hydrogen Energy Materials Korea Institute of Science and Technology Seoul 02792 Republic of Korea; ^3^ School of Mechanical Engineering Yonsei University Seoul 03722 Republic of Korea; ^4^ Department of Chemical and Biological Engineering Korea University Seoul 02841 Republic of Korea; ^5^ Advanced Analysis Center Korea Institute of Science and Technology Seoul 02792 Republic of Korea

**Keywords:** anti‐coking, CO_2_ electrolysis, heterostructure, medium‐entropy materials, SOEC

## Abstract

Conventional solid oxide electrolysis cells (SOECs) with nickel/yttria‐stabilized zirconia (Ni/YSZ) electrodes suffer from low CO_2_ reduction activity and severe carbon deposition below 800 °C, limiting scalability. This study introduces a novel medium‐entropy alloy/Mn‐based oxide composite catalyst deposited via simple infiltration onto the fuel electrode, creating hierarchical heterogeneous metal/oxide nano‐interfaces. The catalyst‐decorated cell achieves a remarkable 46% increase in CO_2_ electrolysis current density, reaching 2.15 A cm^−2^ at 1.5 V and 750 °C. Simultaneously, the catalyst demonstrates exceptional carbon deposition resistance, evidenced by a 75% increase in the current density threshold for carbon formation. The cell maintains stable, carbon‐free operation for 200 h at an extreme current density of 1.0 A cm^−2^. Comprehensive analyses combining in situ characterization and density functional theory (DFT) calculations revealed the enhanced performance originates from synergistic effects between the unique composition of the medium‐entropy alloy and Mn‐based oxides, and their distinctive nanostructured interfaces. This work presents a promising approach for developing advanced electrode materials for CO_2_ electrolysis in SOECs, significantly contributing to the scalability and practical application of this critical technology.

## Graphical abstract

A novel FeCoNiCu/MnO composite heterostructure catalyst is fabricated on a Ni‐based electrode via impregnation, enabling efficient CO2 electrolysis in solid oxide electrolysis cell with a current density over 2 A cm^−2^ at 750 °C and 1.5 V. The cell shows stable operation at 1 A cm^−2^ for 200 h without carbon deposition, offering a scalable strategy for CO_2_ reduction.

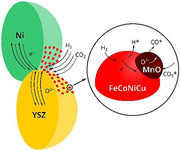

## 1 Introduction

Solid oxide electrolysis cells (SOECs) represent a promising technology for achieving future carbon neutrality goals. They can electrolyze H_2_O and/or CO_2_ to produce H_2_ and/or CO and co‐electrolyze H_2_O/CO_2_ to generate H_2_/CO syngas. At present, demonstration projects with stack capacities exceeding the kilowatt scale have been realized for both H_2_O electrolysis and co‐electrolysis processes.^[^ ^1^ ^]^ However, CO_2_ electrolysis for CO production remains at the laboratory stage due to two major challenges. First, the average bond energy of the C═O bond in CO_2_ molecules is as high as 804.4 kJ mol^−1^,^[^ ^2^ ^]^ necessitating higher temperatures or higher voltages for CO_2_ reduction, which increases the operational costs of the electrolysis cells. Second, and more critically, the CO produced from CO_2_ electrolysis is highly susceptible to carbon deposition or coking via the Boudouard reaction 2CO (g) ⇌ C + CO_2_ (g) below 800 °C.^[^ ^3^ ^]^ This leads to the coverage of catalytic sites, electrode cracking, and other issues that severely hinder sustained operation.^[^ ^4^ ^]^ The fuel electrode of SOECs typically employs a Ni/oxide composite cermet, where carbon deposition on the Ni surface is thermodynamically favorable, promoting rapid degradation of the cell.^[^ ^5^ ^]^ Therefore, achieving cost‐effective CO production from CO_2_ electrolysis in SOEC at lower temperatures is extremely challenging, particularly because of lacking suitable fuel electrode that exhibits simultaneously high catalytic performance toward CO_2_ reduction and good carbon deposition resistance.

One way to address the carbon deposition issue is replacing Ni/oxide cermet with ionic and electronic mixed conducting oxide electrodes such as ceria and perovskite oxides.^[^ ^6^ ^]^ The abundant oxygen vacancies in these materials can enhance CO_2_ adsorption and thus formation of carbonate intermediates at surface, thereby improving CO_2_ electrolysis performance and simultaneously suppressing carbon deposition.^[^ ^7^ ^]^ However, the oxide materials generally have significantly lower electronic conductivities and catalytic properties than Ni.^[^ ^8^ ^]^ Therefore, many efforts have been devoted to developing strategies for enhancing the catalytic activities of oxide systems. A representative example is the in situ formation of metallic nanoparticles anchored at the oxide surface upon a reduction process.^[^ ^9^ ^]^ While this approach has proven to be somewhat effective, it remains challenging to scale up for practical industrial applications.

In this regard, modification of the conventional Ni/oxide cermet to improve its carbon deposition resistance remains a very attractive approach. Recently, application of entropy‐increased alloys, i.e., high and medium entropy alloys, in the fuel electrode materials, benefiting from their “cocktail effect” of abundant catalytic surface sites and the enhance thermodynamic and kinetic stability, shows significant potential in simultaneously improving the catalytic activity and stability of the electrode.^[^ ^10^ ^]^ Inspired by the increased coking tolerance of Ni based binary alloys such as Ni–Cu^[^ ^11^ ^]^ and Ni–Fe,^[^ ^12^ ^]^ a FeCoNiCuAl high‐entropy alloy catalyst was designed to effectively mitigate carbon deposition in solid oxide fuel cells (SOFCs) utilizing methane.^[^ ^13^ ^]^ However, carbon deposition under the SOEC condition is not exactly the same as that resulting from hydrocarbon fuel reforming in SOFC operation, and the effect of high/medium‐entropy alloys on coking in SOECs is unclear. More interestingly, while the nanostructured heterogeneous metal/oxide interface has been shown to be essential for catalytic activities in high temperature CO_2_ electrolysis,^[^ ^14^ ^]^ its role in carbon deposition behavior remains elusive. Furthermore, when it comes to the combination with entropy increased alloys, additional challenge arises from the rational design of alloy and oxide compositions and a controllable way of nano‐interface construction.

In this work, we innovatively designed and fabricated a FeCoNiCu quaternary medium‐entropy alloy/MnO nanocomposite, named as FCNC/MnO, for simultaneous enhancement in the CO_2_ electrolysis activity and carbon deposition resistance. While FCNC has demonstrated high catalytic properties in low‐temperature electrolysis applications,^[^ ^15^ ^]^ MnO is noticed for its good carbon deposition resistance in thermal reforming of methane due to its strong CO_2_ adsorption and carbonate formation,^[^ ^16^ ^]^ which potentially can also improve the catalytic activity in CO_2_ electrolysis due to its low electronegativity.^[^ ^17^ ^]^ More importantly, the nanocomposite was successfully incorporated into the fuel electrode featuring a stable and hierarchically nano‐structured alloy/oxide interface through a straightforward one‐step infiltration of oxide precursor and in situ reduction process, taking advantage of the different thermodynamic stability of metal oxides with respect to their metallic state upon reduction. Following this strategy, remarkable performance was achieved in terms of H_2_ generation power, CO_2_ electrolysis capability, and carbon deposition resistance stability. Underlying mechanisms of the outstanding performance are revealed to correlate with the unique combination of the catalyst composition and its nanostructure in the electrode, which facilitates effective metal and oxygen coupling that not only promotes electrochemical steps for electrolysis but also improves the carbon deposition resistance modified reaction mechanism.

## 2 Result and Discussion

### 2.1 Characterization of FCNC/MnO Nanocomposites

**Figure 1:**
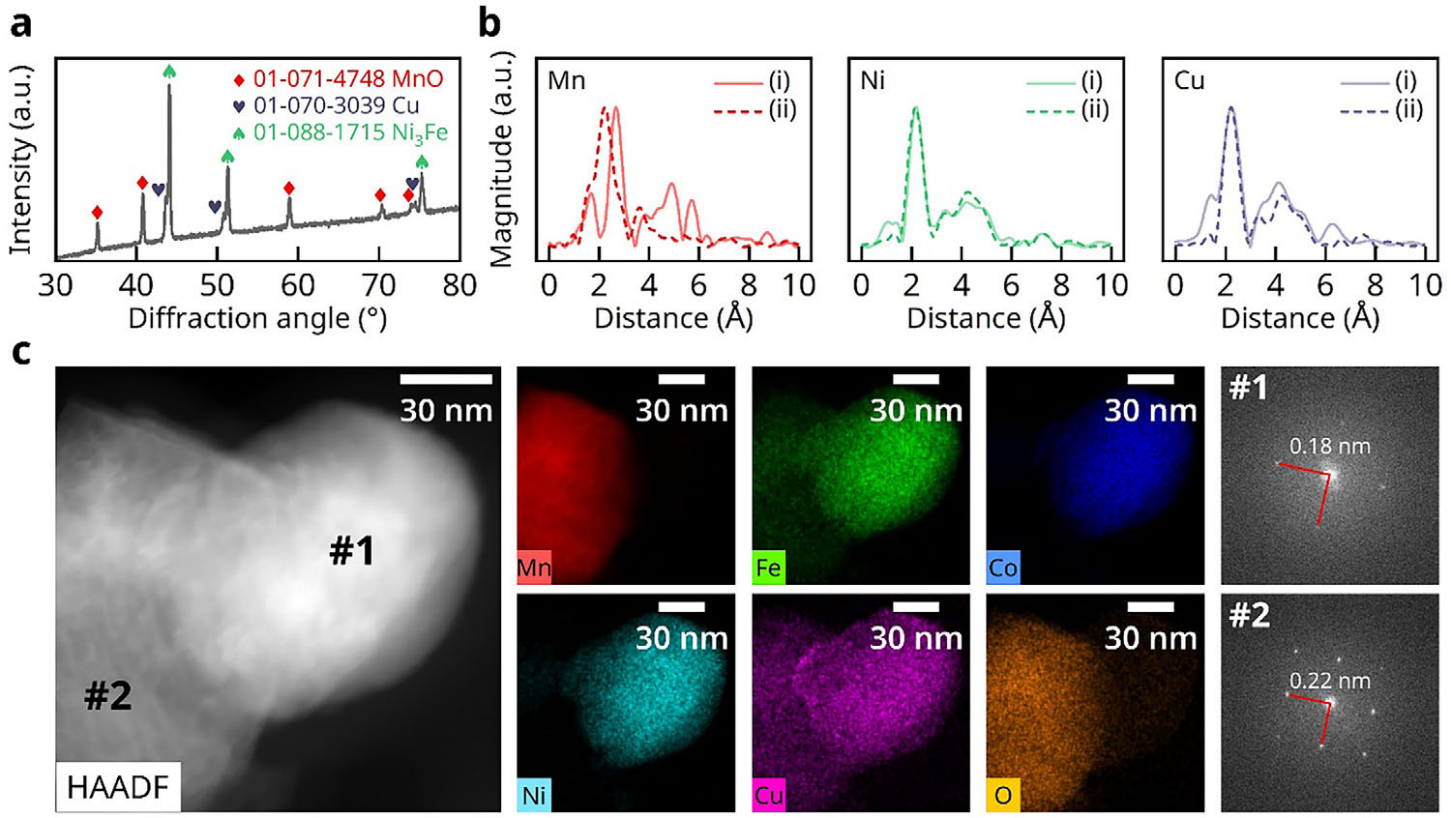
Structural analysis of MCFNC nanocomposites. a) XRD pattern (The label indicates a similar peak position but does not confirm the substance) and b) FT‐EXAFS spectra at Mn, Ni, and Cu K‐edge obtained from (i) FCNC/MnO after reduction at 750 °C and (ii) corresponding foil reference materials. c) HAADF–STEM image and corresponding EDS map and FFT patterns from the marked regions #1 and #2.

Prior to impregnating the FCNC/MnO catalyst precursor solution into the fuel electrode, we first calcined a portion of the precursor solution at 850 °C in an air atmosphere for 1 h, followed by reduction in a 4% H_2_/Ar atmosphere at 750 °C to simulate the formation and transformation process of the catalytic material within the electrode. X‐ray diffraction (XRD) was first performed on the powder sample, and the results are shown in **Figure** **1**a. The diffraction pattern clearly indicates that the mixture primarily consists of three phases. The dominant phase is a multi‐component alloy, whose XRD peak positions closely match those of Ni_3_Fe alloy, with slight peak shifts likely caused by lattice parameter changes due to Co and Cu doping. Additionally, weaker signals corresponding to MnO and Cu were observed. Mn, being a low‐electronegativity element, is difficult to reduce with hydrogen,^[^ ^18^ ^]^ while Cu may have segregated due to its significant lower melting point compared to Fe, Co, and Ni.^[^ ^19^ ^]^ In comparison, the FeCoNiCu quaternary alloy synthesized using the same method exhibits predominantly a single phase metal with a similar presence of slight Cu segregation (Figure S1, Supporting Information).

To further reveal the true chemical states and coordination environments of the elements, synchrotron‐based X‐ray absorption spectroscopy (XAS) experiment was conducted. Figure 1b presents the Fourier‐transformed extended X‐ray absorption fine structure (FT‐EXAFS) signals in R‐space for representative elements Mn, Ni, and Cu. The X‐ray absorption near‐edge structure (XANES) signals for metal elements and additional details are provided in the Figure S2 (Supporting Information). The Mn K‐edge XANES energy is higher than that of Mn foil, indicating an oxidized state of Mn. Additionally, the R‐space data predominantly reveals distinct first peak positions for Mn: for FCNC/MnO, the first nearest neighbor is Mn─O, while for Mn foil, it is Mn–Mn, corresponding to the oxidized and metallic states, respectively. For Fe, Co, Ni, and Cu, the K‐edge absorption edge energy aligns with that of their respective metal foils, confirming their metallic state. The characteristic peaks in R‐space, both in width and position, closely match those of the metal foils, likely due to the minimal differences in atomic radii, such that the alloying process does not introduce significant variations in these features. However, their signals in the R space all appear to exhibit oxide‐related peaks of certain intensity, which may be attributed to the formation of a heterostructure with MnO.

To elucidate the surface chemistry state of catalyst, X‐ray photoelectron spectroscopy (XPS) was performed on FCNC/MnO using a FCNC quaternary alloy as a reference, with results presented in the Figure S3 (Supporting Information). The XPS analysis revealed that Mn predominantly existed in the +2 oxidation state at surface of FCNC/MnO. In addition, after the incorporation of Mn, the peak area ratios of the oxidized states relative to the metallic states for all elements increased, accompanied by a concomitant rise in the proportion of lattice oxygen (Figure S3, Supporting Information). For Co, its zero‐valent state on the surface appeared to vanish, possibly due to electron transfer from Co to Fe and Ni surfaces, which can be evidenced from the shifting in the binding energies of Fe and Ni to lower values, suggesting an increase in electron density on their surfaces. This indicates that introducing MnO in the catalyst promotes surface oxidation of other metallic elements, consistent with the synchrotron radiation test results. Furthermore, electron spin resonance (EPR) experiments on both FCNC/MnO and the FCNC alloy (Figure S4, Supporting Information) confirm that the FCNC alloy contains almost no oxygen vacancies as a result of its pure metallic form, while FCNC/MnO exhibits a pronounced oxygen vacancy signal due to the presence of MnO. Such an increase in oxygen vacancies is generally beneficial for CO_2_ electrolysis.^[^ ^20^ ^]^

Transmission electron microscopy (TEM) was further employed to examine the phase distribution in the composite powder, as shown in Figure 1c. The high‐angle annular dark‐field scanning TEM (HAADF‐STEM) image reveals two distinct particles in regions #1 and #2. The diffraction patterns obtained from Fourier transforms of these regions suggest the presence of two different crystalline phases. The calculated lattice spacings of 0.18 and 0.22 nm closely match the (200) and (020) planes of the alloy (which is similar to Ni_3_Fe) and the MnO phases, respectively. Energy‐dispersive X‐ray spectroscopy (EDS) mapping shows significant overlap in the distributions of Fe, Co, Ni, and Cu, while Mn and O distributions are consistent, indicating the presence of FCNC and MnO primary phases. Based on EDS quantification (after excluding trace amounts of O and Mn elements), the alloy phase consists of Fe (28 at.%), Co (30 at.%), Ni (28 at.%), and Cu (14 at.%). The calculated configurational entropy of the quaternary alloy is 1.35 R, which meets the criterion for medium‐entropy alloys.^[^ ^21^ ^]^ A very small amount of O of 4.7 at.% is also noticed in the Fe, Co, Ni, Cu overlapping region, likely resulting from the surface oxidation of the alloy in consistent with the XPS analysis. Additionally, Cu and Fe exhibit partial overlap with O, suggesting some diffusion of these two elements into MnO forming (Mn,Cu/Fe)O*_x_*, likely due to their relatively lower melting points and higher stability of their oxides.^[^ ^22^ ^]^ Meanwhile, low‐magnification TEM observations revealed slight Cu segregation, consistent with the XRD results (Figure S5, Supporting Information). Nevertheless, XANES and EXAFS signals indicate that the majority of Fe and Cu remain in the reduced alloy in a metallic state. Collectively, these characterization techniques confirm that the catalyst consists of mutually loaded FCNC and MnO based phases, forming a medium‐entropy alloy/oxide nanocomposite with the alloy surface exhibiting a certain degree of oxidation.

### 2.2 Infiltration Process

**Figure 2:**
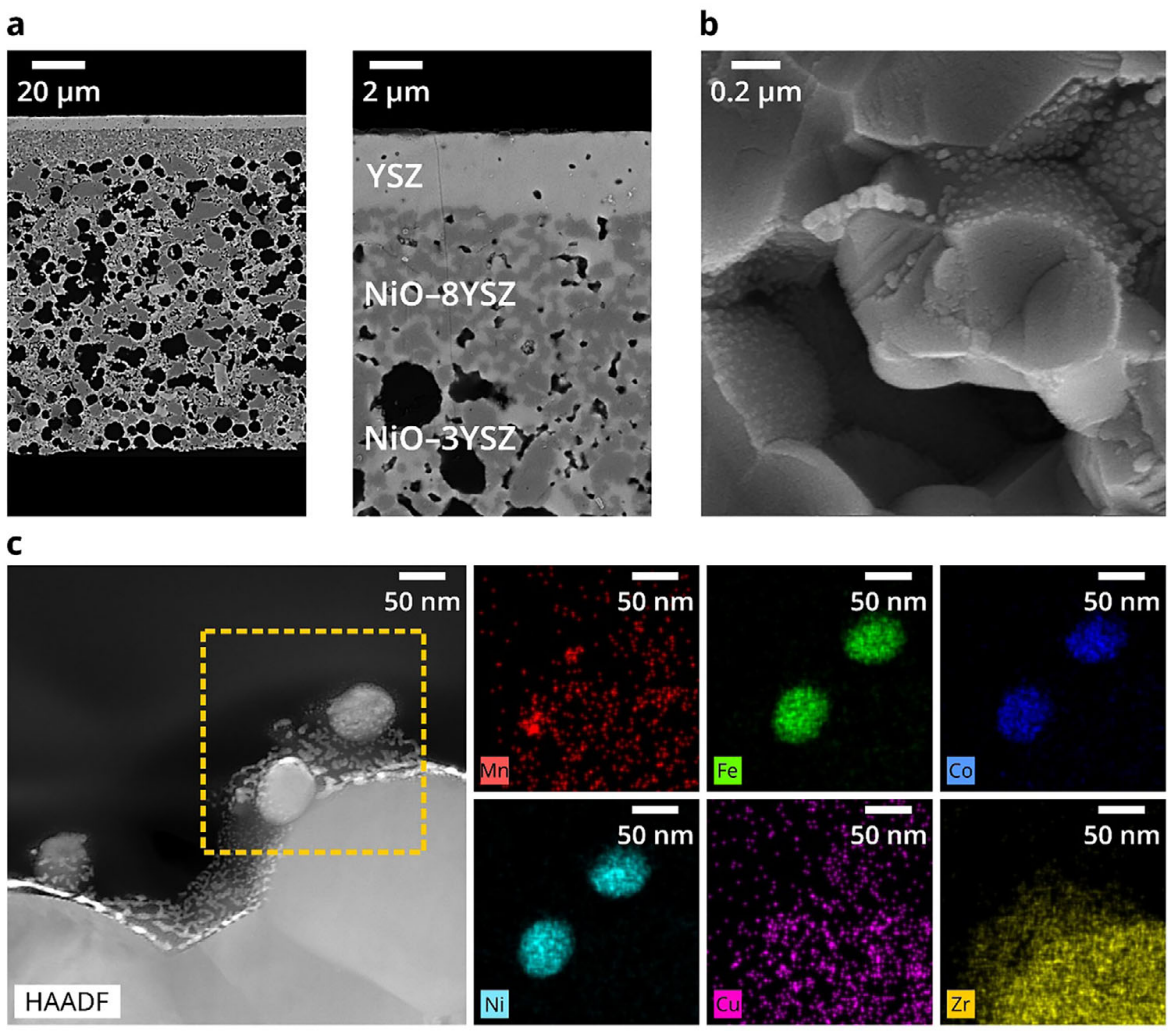
Characteristics of infiltrated FCNC/MnO nanoparticles into the fuel electrode. a) Cross‐sectional SEM images of the ultra‐thin fuel electrode support with the NiO–YSZ/YSZ structure. b) SEM image of the functional layer after infiltration with FCNC/MnO catalyst. c) HAADF‐STEM image and corresponding EDS map obtained from FIB sectioning of the fuel electrode.

In developing novel electrode catalysts for SOECs, wet infiltration is widely employed because of its simplicity and high accessibility.^[^ ^23^ ^]^ Entailing the introduction and thermal decomposition of a precursor solution in the porous structure, the wet infiltration technique often achieves significant improvement in cell performance with minimal catalyst usage. Moreover, it is highly beneficial in the stable electrode formation, considering that newly developed materials often suffer from delamination from the electrolyte after high‐temperature sintering (> 900 °C) due to thermal expansion mismatch.^[^ ^24^ ^]^ The effectiveness of the introduced nano‐catalysts is closely related to the amount of catalyst distributed within a very thin region near the electrolyte‐electrode interface. However, traditional fuel electrode‐supported cells typically feature a thick support layer (usually over 300 µm) to ensure mechanical strength,^[^ ^25^ ^]^ which poses a challenge for the precursor solution to penetrate the functional layer solely by capillary effect. Additionally, a significant portion of the precursor solution remaining in the support layer leads to waste. To address this, we employed a self‐developed ultra‐thin fuel electrode‐support cell, as shown in the scanning electron microscopy (SEM) image in **Figure** **2**a. This cell before reduction consists of a ≈120‐µm‐thick NiO/(Y_2_O_3_)_0.03_(ZrO_2_)_0.97_ (3YSZ) support and a ≈20‐µm‐thick NiO/(Y_2_O_3_)_0.08_(ZrO_2_)_0.92_ (8YSZ) fuel electrode functional layer, providing sufficient mechanical strength for the following fabrication process (e.g., screen printing) and electrochemical testing.^[^ ^26^ ^]^ After impregnating the catalyst solution into the fuel electrode and subjecting it to a heat treatment similar to that of the powder sample, numerous nanoscale particles were uniformly distributed on the internal pore scaffold of the functional layer, as shown in Figure 2b, confirming the successful infiltration process. Subsequently, the cell was reduced by H_2_, and the SEM images of the cells with both the non‐impregnated and impregnated catalysts are presented in Figure S6 (Supporting Information).TEM specimens were extracted from the H_2_ reduced electrode using a focused ion beam (FIB) system for further confirmation. Figure 2c shows the HAADF‐STEM image of an FIB‐cut sample, where three catalyst particles with sizes ≈ 50 nm are observed on the YSZ scaffold support. Some continuous flocculent structures on the sample surface are attributed to Pt coating during FIB preparation (EDS result is shown in Figure S7, Supporting Information). EDS analysis of the particles within the yellow dashed box in Figure 2c reveals that Fe, Co, Ni, Mn, and Cu co‐exist in a same area, but the signal intensities of Mn and Cu are weaker compared to the other elements. At the same time Mn and Cu show additional co‐aggregation with O element (The EDS map of O element almost coincides with that of Zr element, as shown in Figure S8, Supporting Information). Notably, partial distributions of Mn and Cu are detected in the YSZ scaffold, suggesting that these elements may have diffused into the YSZ lattice. This is reasonable given that Mn is inherently difficult to reduce and may thus remain in the YSZ lattice,^[^ ^22^ ^]^ while Cu, potentially due to its lower melting point or its valence states transformation, facilitates its easier diffusion into the lattice and subsequent integration with YSZ.^[^ ^27^ ^]^ The incorporation of these elements into YSZ may also enhance the lattice oxygen activity.^[^ ^28^ ^]^ Meanwhile, nanoparticles similar to those observed on the YSZ surface were also detected on the Ni surface, with some dispersed near the interface between the two materials, suggesting an extension of the triple‐phase boundaries (TPBs), as illustrated by the SEM–EDS results in Figure S9 (Supporting Information).^[^ ^29^ ^]^ Regardless, these results confirm the successful infiltration of the catalyst with a similar phase composition and distribution as that observed in the powder sample. However, after the infiltration and in situ reduction process, a hierarchical structure containing multiple types of heterogeneous metal/oxide nano‐interfaces is formed. These include, first, the interfaces between the YSZ scaffold and FCNC quaternary alloy nanoparticles, and second, the interfaces between (Mn,Cu)O*_x_* oxide nanoparticles grown in situ on the alloy surface.

### 2.3 Electrochemical Performance Measurement

**Figure 3:**
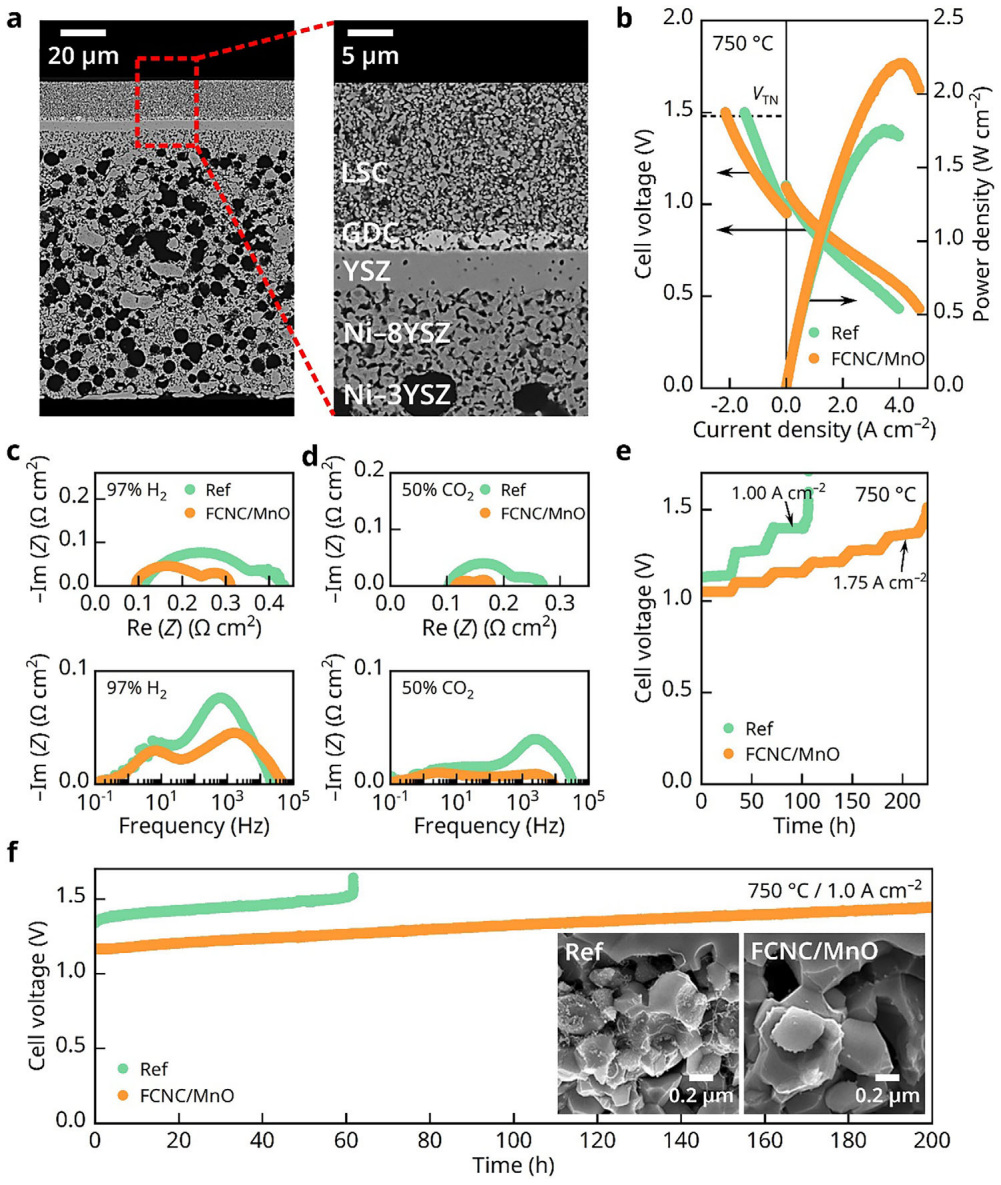
Structural and electrochemical characteristics of FCNC/MnO‐infiltrated cell. a) Cross‐sectional SEM images of the tested cell. b) *i*–*V*–*P* curves and (c–d) Nyquist and Bode plots of EIS under OCV conditions in (c) fuel cell and (d) electrolysis modes at 750 °C with and without FCNC/MnO infiltration. e) Evaluation of threshold electrolysis current densities and f) long‐term stability tests at 1.0 A cm^−2^ at 750 °C with 50% CO_2_/CO and postmortem analysis of the fuel electrodes (inset).

We then electrochemically evaluated a full cell impregnated with FCNC/MnO nano‐catalysts and compared it with a bare cell with the Ni–YSZ/YSZ/Gd_0.1_Ce_0.9_O_2–δ_ (GDC)/La_0.6_Sr_0.4_CoO_3–δ_ (LSC) structure (**Figure** **3**a). As shown in Figure 3b, near‐theoretical open‐circuit voltages (OCVs) were obtained, indicating gas tightness of the YSZ electrolyte and glass sealant. When operated at 750 °C, the FCNC/MnO cell achieved a peak power density of 2.21 W cm^−2^ in the SOFC mode (H_2_ power generation), a 26% improvement over the reference cell's 1.76 W cm^−2^. In the SOEC mode (CO_2_ electrolysis), the improvement was more pronounced, with the FCNC/MnO cell achieving an impressive current density of 2.15 A cm^−2^ at 1.5 V, a 46% increase over the reference cell's 1.47 A cm^−2^. Both SOFC and SOEC performance are much better than those reported for Ni–YSZ fuel electrode‐supported cells with similar electrolyte and air electrode operating around 750 °C and approaching those using more active electrolyte and air electrode materials (see Tables S1 and S2, Supporting Information for data comparison).

The electrochemical impedance spectroscopy (EIS) test results of the FCNC/MnO and the reference cell are shown in Figure 3c,d. In SOFC mode, the resistance decreased from 0.43 to 0.31 Ω cm^2^, a reduction of ≈28%. The reduction was even greater in CO_2_ electrolysis mode, where the resistance dropped from 0.27 to just 0.18 Ω cm^2^, a 33% decrease. Analysis of the Bode plot revealed that the reduction in impedance was most significant in the mid‐to‐high frequency range (> 100 Hz), corresponding to the electrochemical reaction process, while the low‐frequency diffusion impedances were nearly identical for both cells, collectively indicating that the impregnated catalyst significantly accelerated the reaction rates of H_2_ utilization and CO_2_ reduction without causing additional resistance to gas diffusion.

The carbon deposition resistance of the cell during CO_2_ electrolysis is another critical evaluation parameter. As our previously reported,^[^ ^30^ ^]^ when the current density exceeds a critical threshold, the electrolysis voltage of the cell suddenly surges, leading to rapid failure within minutes or even seconds. This phenomenon serves as a signal for carbon deposition, often accompanied by severe and irreversible damage, such as extensive carbon buildup in the fuel electrode functional layer and delamination from the electrolyte. Our test results, as shown in Figure 3e, evaluate the stability of two cells by incrementally increasing the current density from 0.5 A cm^−2^, a common value in industrial electrolysis, in steps of 0.25 A cm^−2^, with each step maintained for 30 min. The reference cell exhibited a sudden voltage surge and failure when the current density exceeded 1.0 A cm^−2^. In contrast, the FCNC/MnO cell remained relatively stable even at a high current density of 1.75 A cm^−2^, with a voltage surge only occurring at higher current densities. This indicates that the critical current density threshold for carbon deposition resistance in the FCNC/MnO cell was increased by ≈75%. To further analyze the impact of carbon deposition, the impedance of the cells before and after failure was compared (see Figure S10, Supporting Information for the FCNC/MnO cell example). The Nyquist plot shows a significant increase in polarization resistance after carbon deposition, with the Bode plot revealing a notable increase in impedance at high frequencies and no significant change in the low‐frequency mass transport region. This can be well attributed to carbon deposition covering the catalytic sites, reducing the number of TPBs.

### 2.4 Long‐Term Stability in Carbon Deposition Resistance

The long‐term carbon deposition resistance of the FCNC/MnO cell was further evaluated by using an accelerated condition at an extremely high current density of 1.0 A cm^−2^ for 200 h, with the reference cell as the control group. As shown in Figure 3f, the reference cell exhibited a sudden voltage surge due to carbon deposition after ≈60 h, while the FCNC/MnO cell showed no such behavior throughout the entire test. A comparable degradation rate during the steady‐state stage was observed in both cells. Postmortem analysis of the operated cell reveals that the observed degradation is mostly attributed to the decomposition of the LSC oxygen electrode and Cr poisoning from the metal fixtures, resulting in the possible formation of SrO, Co_3_O_4_ particles and insulating Sr─Cr oxides dense structures (Figures S11 and S12, Supporting Information),^[^ ^30^, ^31^ ^]^ which can lead to increase in cell resistance, oxygen buildup at the oxygen electrode and electrolyte interface and consequent delamination due to blocking of gas transport.^[^ ^32^ ^]^

The stark difference in carbon deposition in the fuel electrode between reference and FCNC/MnO cells after operation can be seen in Figure 3f. Numerous carbon fibers were observed in the functional layer near the electrolyte side of the reference cell, while no such structures were detected in the FCNC/MnO cell. Additionally, Raman spectroscopy was performed on the cross‐section of the functional layer near the electrolyte. The Raman spectrum in Figure S13 (Supporting Information) reveals a prominent carbon peak in the reference cell, but no carbon signal was detected in the FCNC/MnO cell. These analyses collectively demonstrate that the FCNC/MnO medium‐entropy catalyst exhibits excellent carbon deposition resistance, effectively preventing carbon formation during electrolysis.

The high durability of the FCNC/MnO cell against carbon deposition is closely related to the stability of the catalyst particles. As shown in Figure S14 (Supporting Information). the particles distributed on the electrode scaffold remained uniform, with sizes ≈ 50 nm, showing no significant agglomeration or growth compared to the pre‐stability test. This indicates that the catalyst particles were stably anchored on the YSZ scaffold. Further EDS analysis (Figure S15, Supporting Information) demonstrates similar composition of the catalyst to those before electrolysis, with the five elements (Mn, Fe, Co, Ni, Cu) still closely integrated, maintaining the nanocomposite structure of the FCNC quaternary alloy and MnO particles without additional segregation of elements.

### 2.5 Mechanisms of Enhanced Performance

Based on the microstructural investigation, the FCNC/MnO incorporated fuel electrode exhibits a unique feature with the presence of multiple metal and oxide phases and hierarchical heterogeneous interfaces, where the in situ reduction induced Mn based oxide is first closely integrated with FCNC phase forming a nanocomposite, and second this composite exists as nanoparticles anchoring at the YSZ scaffold surface.

To elucidate whether a synergetic effect from these multiple phases and associated heterogeneous interfaces exists for the enhanced electrochemical activity and carbon deposition resistance, full cell tests were further carried out on two additional control groups: cells impregnated with either the FCNC quaternary alloy or MnO nanoparticles and the results are shown in Figure S16 (Supporting Information). Notably, both catalysts exhibited inferior performance in both SOFC and SOEC modes compared to the FCNC/MnO one, confirming the co‐operative effect of the dual phase in the FCNC/MnO catalyst. Interestingly, comparing within these two cells, the FCNC‐impregnated cell showed better performance in SOFC mode but worse in SOEC mode, while the MnO‐impregnated cell exhibited the opposite trend. The FCNC‐impregnated cell achieved a maximum power density of 1.95 W cm^−2^, an 11% improvement over the reference cell, while the MnO‐impregnated cell showed almost no improvement at 1.8 W cm^−2^. This indicates that the FCNC alloy phase is more suitable for catalyzing H_2_ oxidation than the MnO oxide phase. In SOEC, the FCNC‐impregnated cell achieved a current density of only 1.65 A cm^−2^ at 1.5 V, while the MnO‐impregnated cell achieved a remarkable current density of 2.04 A cm^−2^, suggesting that the CO_2_ electrolysis performance enhancement in the FCNC/MnO may primarily attributed to the MnO oxide phase. However, it should be noted that the MnO content in the MnO‐impregnated cell was five times higher than that in the FCNC/MnO cell.

The beneficial effect of MnO incorporation into FCNC was confirmed by H_2_‐temperature‐programmed desorption (H_2_‐TPD) and CO_2_‐TPD experiments, as shown in Figure S17 (Supporting Information). In the H_2_‐TPD results, the main desorption peak for FCNC/MnO appears at ≈ 320 °C, compared to ≈280 °C for FCNC, suggesting a slightly enhanced H_2_ binding capability of FCNC/MnO. In the CO_2_‐TPD experiment, both materials exhibit similar behavior below 300 °C. However, FCNC/MnO exhibits two distinct desorption peaks at approximately 400 and 550 °C, corresponding to the decomposition of bidentate and monodentate carbonates,^[^ ^33^ ^]^ respectively. In contrast, the FCNC alloy shows only a single prominent peak around 600 °C, primarily associated with monodentate carbonate species.^[^ ^33^ ^]^ These results indicate that FCNC/MnO possesses more favorable CO_2_ adsorption behavior, which may contribute to its improved catalytic performance.

Furthermore, the synergetic effect of FCNC/MnO can also be seen on the carbon deposition resistance (shown in Figure S18, Supporting Information). Although both catalyst‐impregnated cells showed improvements in the critical current density threshold for carbon deposition, achieving 1.25 A cm^−2^ for FCNC and 1.5 A cm^−2^ for MnO‐impregnated cells, these values are still lower than 1.75 A cm^−2^ of the FCNC/MnO cell. In addition, during a 100‐h electrolysis test at 1.0 A cm^−2^, the MnO cell exhibited a degradation rate ≈ 1.3 times of the FCNC/MnO cell (210 mV/100 h for MnO vs 160 mV/100 h for FCNC/MnO, as shown in Figure S19, Supporting Information).

**Figure 4:**
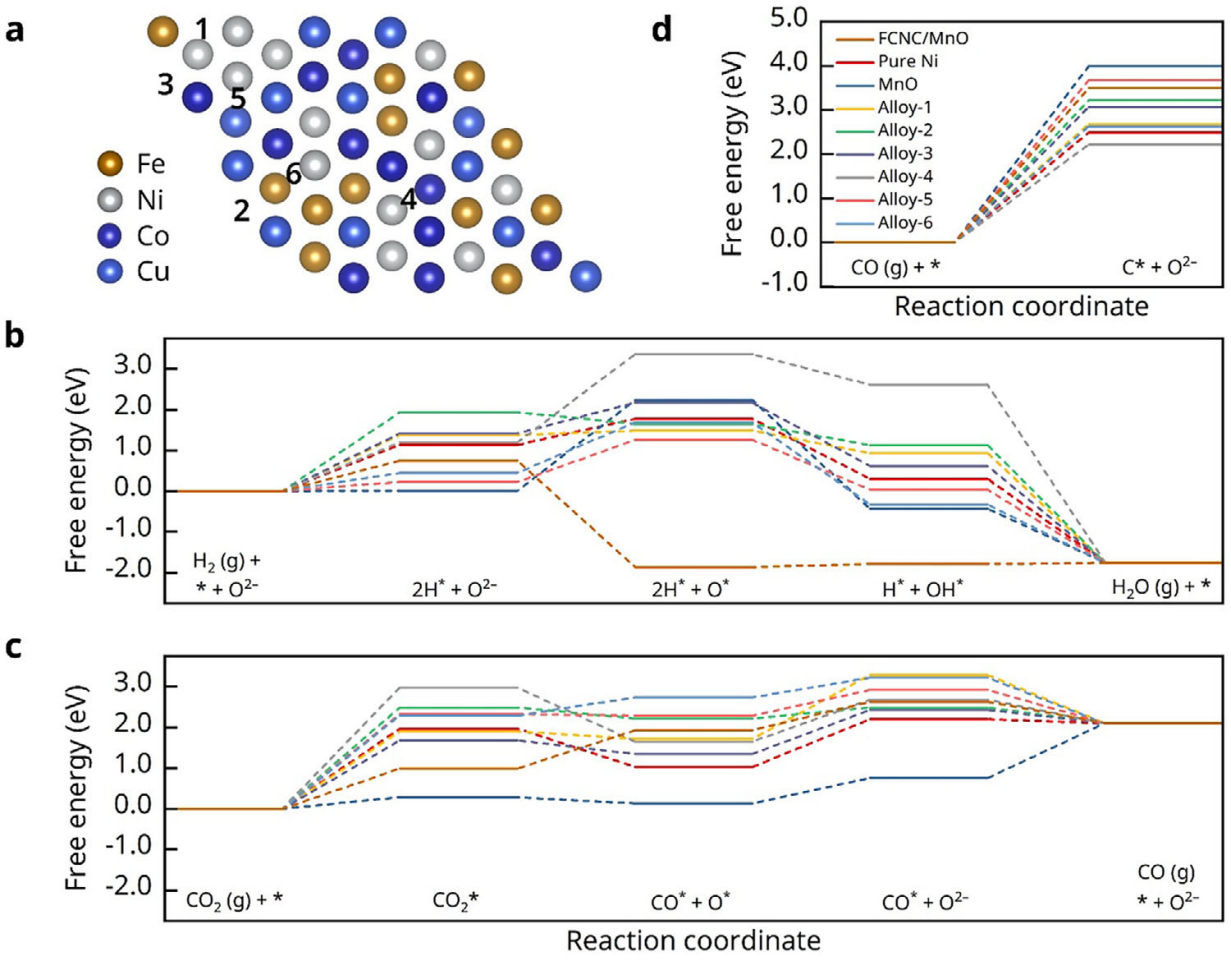
DFT calculation results. a) Calculation model of FCNC and the selected surface sites; b–d) represent the Gibbs free energy changes for each step of H_2_ conversion to H_2_O, CO_2_ reduction to CO, and the CO cracking reaction to form coke on different surface sites, respectively.

The above experimental investigation clearly reveals that the FCNC/MnO nanocomposite system exhibit a synergetic effect that combines and maximizes the benefits of the individual components. DFT calculations was conducted to further elucidate the underlying mechanism in the role of the different component. Based on the experimental results, we constructed MnO, FCNC, and FCNC/MnO models, using Ni metal as a reference (all models for calculation are provided in the Figure S20, Supporting Information). **Figure** **4**a shows the selected active sites on the FCNC alloy surface with selected active sites, while the sites for other models are shown in the Figure S21 (Supporting Information). The energy barriers for each step of the electrocatalytic process were analyzed. The calculation results for the catalytic conversion of H_2_ to H_2_O in SOFC mode on all materials are presented in Figure 4b. For pure Ni metal, the rate‐determining step (the step requiring the highest energy) in the catalytic conversion of H_2_ is the first step, namely the dissociative adsorption of H_2_ molecules on the Ni surface, which requires an energy barrier of 1.14 eV. In contrast, the dissociative adsorption energy barriers at site 5 on the alloy surface are significantly reduced to 0.23 eV. Meanwhile, the rate‐determining step at site 5—the oxidation of oxide ions (O^2–^) at the interface to form adsorbed oxygen species (O) on the catalyst surface—requires an energy barrier of 1.03 eV, which is also lower than that of the rate‐limiting step on the Ni surface. Other sites also exhibit a significant promoting effect in certain intermediate steps. This indicates that the medium‐entropy alloy optimizes the surface electronic structure by providing multiple combination possibilities of the metal elements, enhancing H_2_ conversion the overall reaction process, thereby improving the power density in SOFC mode. Notably, while the dissociative adsorption energy of H_2_ on the MnO surface is relatively low, the subsequent step becomes the rate‐determining step with an energy barrier of 2.23 eV, which is significantly higher than that on the Ni surface. Therefore, MnO is not considered to be an effective catalyst for promoting H_2_ utilization, as confirmed by the experimental results showing almost no improvement in H_2_ power generation after infiltration. Finally, for the site on the FCNC/MnO model, the highest energy required during the reaction process is only 0.75 eV, which is significantly lower than that at any site on the other model surfaces, clearly demonstrating the advantage of the heterostructure of the composite.

For the CO_2_ reduction process, extensive literature suggests that FCNC also positively influences CO_2_ conversion.^[^ ^34^ ^]^ As shown in Figure 4c, the energy barrier for CO_2_ molecule adsorption on Ni, which is the rate‐determining step, is 1.97 eV. On the FCNC surface, site 3 exhibits an energy barrier of only 1.68 eV, while site 1 shows a barrier of 1.90 eV. Moreover, these catalytically favorable sites are primarily located near Ni, Fe, and Co atoms; therefore, the reduced Cu content after impregnation is likely to have a limited impact on the catalytic performance. Notably, these barriers are further reduced significantly on MnO, reaching as low as 0.29 eV. This indicates that CO_2_ conversion is more favorable on the MnO surface, making it very easy for CO_2_ to adsorb and participate in subsequent reduction reactions.^[^ ^35^ ^]^ For MnO, the rate‐determining step becomes the desorption of CO, with an energy barrier of 1.35 eV, which is still lower than that of the rate‐determining step on other sites. These advantageous conditions explain the enhanced CO_2_ electrolysis performance of MnO. At the FCNC/MnO surface, this energy barrier is further reduced to 0.99 eV, once again demonstrating the synergistic effect of the heterojunction. Next, we investigated the carbon deposition resistance of two components, as shown in Figure 4d. The energy barrier for CO molecule dissociation to form carbon on the Ni surface is 2.49 eV, indicating that this process may occur at the certain electrolysis voltage. However, the energy barrier for carbon dissociation on the MnO surface is as high as 4.00 eV, much larger than that on the Ni surface, meaning MnO significantly inhibits the further dissociation of CO into carbon. FCNC/MnO requires an energy of 3.5 eV for this step, which likewise enhances its resistance to carbon deposition. Meanwhile, several sites on the alloy surface exhibit CO dissociation barriers that are higher than those of pure Ni, indicating that the use of the alloy can also help suppress carbon deposition.

**Figure 5:**
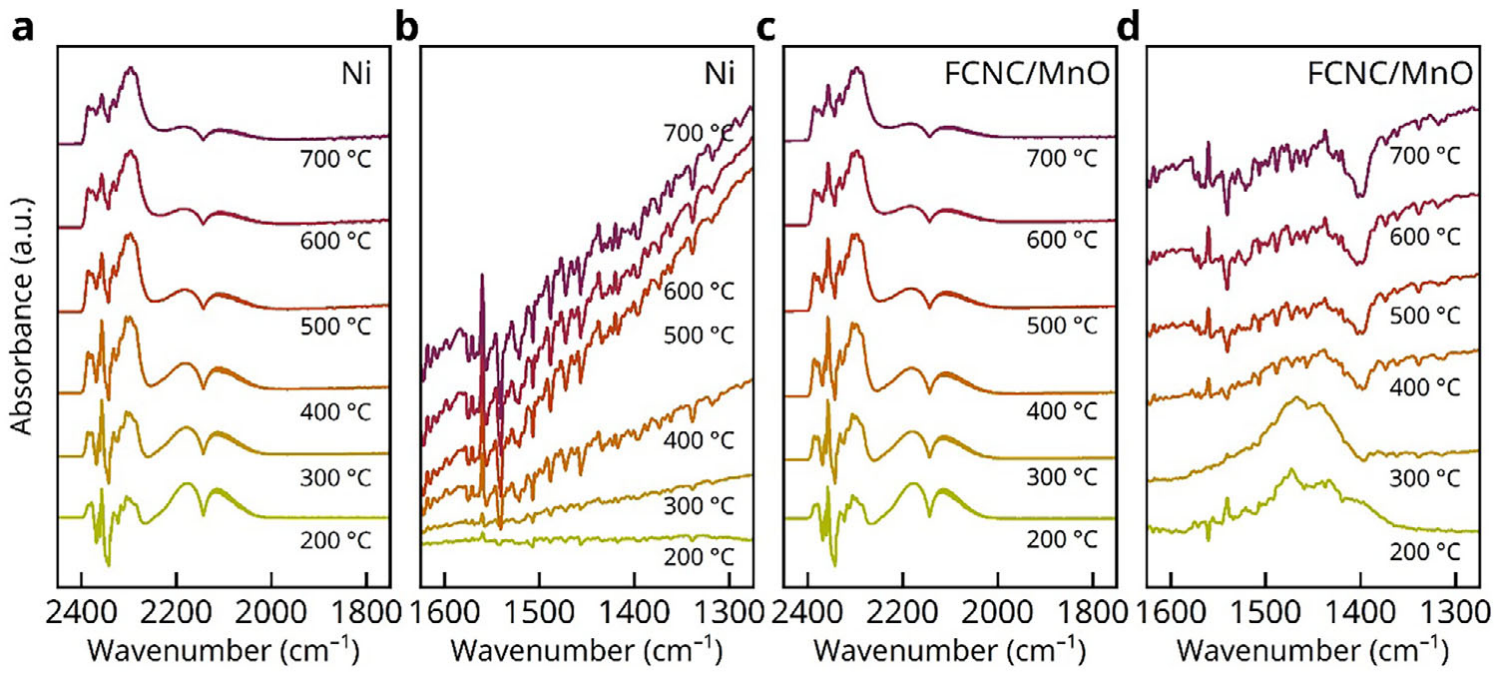
In situ DRIFT investigation. DRIFT spectra of Ni ((a) and (b)) and FCNC/MnO ((c) and (d)) powders measured under a 5% CO–5% CO_2_–90% Ar atmosphere.

Finally, we performed in situ diffuse reflectance infrared Fourier transform spectroscopy (DRIFTS) measurements from 200 to 700 °C. Consistent with the actual experimental conditions, a CO–CO_2_ mixed gas atmosphere was used to prevent sample oxidation. The results are presented in **Figure** **5**. The signals observed in the 2250–2400 cm^−1^ range correspond to molecular vibrations of CO_2_, while those in the 2000–2250 cm^−1^ range are attributed to CO vibrations. Additionally, the signal between 1350–1550 cm^−1^ is typically assigned to carbonate species formed from adsorbed CO_2_ conversion.^[^ ^36^ ^]^ Comparing the infrared spectra of Ni and the FCNC/MnO material, it is evident that no carbonate signals are detected on the metallic Ni surface throughout the temperature range. In contrast, the FCNC/MnO surface exhibits distinct carbonate adsorption signals, which can be attributed to the presence of MnO enhancing CO_2_ adsorption on the metal surface. This observation is consistent with previous reports in the literature and aligns well with the DFT‐predicted results shown in Figure 4c.^[^ ^14^ ^]^ As the temperature increases, the intensity of the carbonate signals progressively decreases. This is partly due to the decomposition of adsorbed MnCO_3_ above 400 °C,^[^ ^37^ ^]^ rendering it undetectable by infrared spectroscopy. Additionally, the relatively low CO_2_ concentration of 5% used in this study may also contribute to the diminishing signals. Nevertheless, the experimentally confirmed stronger CO_2_ capture capability on the FCNC/MnO surface suggests that the surface‐enriched carbonates can provide favorable conditions for accelerated CO_2_ reduction and facilitate the removal of transiently deposited carbon through a modified reaction pathway, distinct from that of Ni, as proposed by Skafte et al.^[^ ^6a^ ^]^ thereby enhancing resistance to carbon deposition from a kinetic perspective.

**Figure 6:**
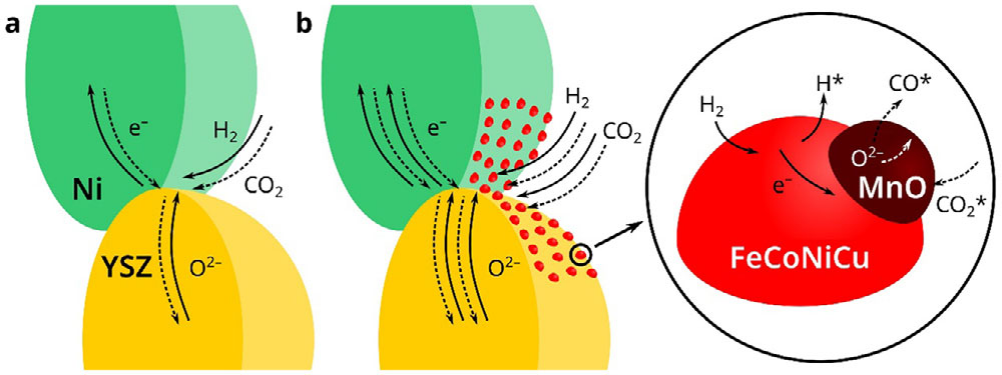
Schematic illustration of the hierarchical structure on the fuel electrode.

In summary, the comprehensive experimental and theoretical studies reveals that the enhanced electrolysis performance and carbon deposition resistance of the FCNC/MnO fuel electrode stems from the synergetic effect based on a unique combination of chemistry and structure optimization (**Figure** **6**). The diverse catalytic sites on the multi‐component alloy surface facilitate efficient H_2_ conversion as well as the CO_2_ electrolysis, the introduction of oxide is particularly important in boosting electrolysis capability and eliminating carbon deposits. Meanwhile, the unique electrode microstructure is considered to be crucial. Combining the high oxygen ion conduction of YSZ, different catalytic property and carbon deposition resistance of FCNC and Mn based oxide, this type of nanostructured interfaces can induce strong coupling of oxygen vacancies in the oxide with the adjacent metal phase, leading to shortened metal‐oxygen and metal‐carbon bonding thus facilitating the required electron transfer and oxygen vacancy incorporation during efficient CO_2_ electrolysis.^[^ ^38^ ^]^ Furthermore, the anchoring of the nanocomposite particles at the YSZ scaffold surface and the in situ redox induced Mn based oxide integrated with the FCNC alloy enable formation of stable interfaces, providing an optimum nanostructure to ensure a durable co‐operation of the different components for CO_2_ electrolysis and carbon deposition resistance.

## 3 Conclusion

In this study, we designed and implemented a novel FCNC/MnO nanocomposite catalyst for high‐temperature CO_2_ electrolysis in SOECs. Through a one‐step infiltration, thermal decomposition of the precursor, and subsequent in situ reduction to form the active catalyst, nano‐structured composite particles comprising the FCNC medium‐entropy alloy phase closely integrated with Mn based oxide one was formed and further anchored at the YSZ fuel electrode surface. This approach significantly enhanced the efficiency of CO_2_ electrolysis with an impressive current density of 2.15 A cm^−2^ at 1.5 V, achieving performance levels at the highest values reported in the literature. Furthermore, the cell exhibited a nearly 75% enhancement in the current density threshold for carbon deposition and maintained coking‐free operation for 200 h under an extreme current density of 1.0 A cm^−2^, showcasing exceptional resistance to carbon deposition. Comprehensive multi‐scale characterization and DFT calculation revealed that the greatly enhanced performance is related to the synergetic effect of the medium‐entropy alloy and the Mn based oxides, which not only relies on the distinct benefit of the composition of the metal and alloy components, but also their unique nanostructure in the electrode, featuring with the presence of hierarchical metal/oxide heterogeneous interfaces, which are electrochemically active and structurally stable at the same time. This work provides a novel approach for improving SOEC performance and offers valuable insights into the development of catalytic materials for high‐temperature CO_2_ electrolysis.

## 4 Experimental Section

### Cell Preparation and Infiltration Process

The fabrication procedure for fuel electrode‐supported cells followed the methodology outlined in our previous work, detailed described in the supporting information.^[^ ^26^ ^]^ All impregnated catalysts were prepared by dissolving the corresponding metal nitrates in anhydrous ethanol, with the total concentration of metal cations maintained at 0.5 mol L^−1^. Equimolar amounts of ethylene glycol and citric acid were added relative to the metal cations. All chemicals were procured from Sigma‐Aldrich. Following the preparation of the solution, 120 µL of the solution was impregnated into the fuel electrode, which was subsequently calcined at 850 °C for 1 h to obtain the impregnated cell.

### Electrochemical Performance Testing

The experimental setup and fixture operations for the cell performance tests were conducted following the methodology detailed in our previous work.^[^ ^30^ ^]^ All tests were performed at 750 °C. For the power generation and CO_2_ electrolysis tests, gas mixtures of 97% H_2_–3% H_2_O and 50% CO_2_–50% CO were respectively fed to the fuel electrode. The air electrode was supplied with air for both modes, with a flow rate of 200 sccm maintained for all gases.

### Material Characterization

Material characterization was performed using the following instruments: X‐ray diffraction (XRD) analysis was conducted on a Bruker D8 Advance diffractometer (Bruker Corp., USA); field‐emission scanning electron microscopy (FE‐SEM) was carried out using an FEI Inspect F microscope (FEI Company, USA); transmission electron microscopy (TEM) was performed on a Talos microscope (Thermo Fisher Scientific Inc., USA); Raman spectroscopy was conducted using a Renishaw inVia confocal Raman microscope (Renishaw plc, UK); EPR measurements were conducted using a Bruker A300 spectrometer (Bruker, Germany); X‐ray photoelectron spectroscopy (XPS) was conducted using a PHI 5000 VersaProbe system (ULVAC‐PHI Inc., Japan) equipped with a monochromated Al Kα X‐ray source (*hυ* = 1486.6 eV); H_2_‐TPD and CO_2_‐TPD experiments were conducted using a Micromeritics AutoChem II 2920 chemisorption analyzer (Micromeritics, USA); and synchrotron radiation XAS experiments were performed at the 10D‐KIST beamline of the Pohang Accelerator Laboratory (PAL, South Korea).

### In Situ DRIFTS Measurements

In situ DRIFTS were performed using Nicolet IS50 FTIR instrument (Thermo Fisher Scientific) equipped with DiffusIRTM (PIKE Technologies). Spectra were obtained with 64 scans with a resolution of resolution of 4 cm^−1^ through a Mercury‐Cadmium‐Telluride (MCT) detector. For sample preparation, 10 wt% of the sample powder was diluted with Potassium bromide (KBr) powder, ground, and evenly loaded into a porous ceramic cup. Before analysis, the samples in the DRIFTS chamber were reduced in the 10% H_2_/Ar mixture (30 mL min^−1^) at 600 °C for 1 h. The samples were then increased to 700 °C and purged with Ar gas (30 mL min^−1^) for 30 min to remove any adsorbed H_2_ from the reduction process. During cooling, background spectra were recorded at temperatures of 700 to 200 °C with interval of 100 °C for each sample. Once cooled to 25 °C, the samples were exposed to 5% CO–5% CO_2_–90% Ar gas (30 mL min^−1^) for 30 min to reach saturation. Spectra were subsequently recorded at 200 to 700 °C with interval of 100 °C under continuous reaction gas flow. The measurements were taken at each point for 30 min to get steady state data.

### DFT Calculation

In this work, all theoretical computations based on spin‐polarized density functional theory (DFT)^[^ ^39^ ^]^ were conducted by Vienna Ab initio Simulation Package (VASP).^[^ ^40^ ^]^ The ion‐electron interactions were treated by projector augmented wave (PAW) method.^[^ ^41^ ^]^ Generalized gradient approximation (GGA)^[^ ^42^ ^]^ of the Perdew‐Burke Enzerhor (PBE) functional^[^ ^43^ ^]^ was used to describe the exchange‐correlation function. The plain‐wave cutoff energy was set to 450 eV and DFT‐D3 scheme^[^ ^44^ ^]^ was employed to consider the long‐range van der Waals correction. The first Brillouin zone was sampled by 3 × 3 × 1 Monkhorst‐Pack scheme k‐point mesh according to the feature of crystalline lattice. The convergence criteria of energy and force for all structural optimization and energy calculation were set to 10^−4^ eV and 0.03 eV Å^−1^, respectively. With respect to the performance assessment, reaction Gibbs free energy *G* was calculated by *ΔG = ΔE + ΔZPE – TΔS* (*E*: total energy, *ZPE*: zero‐point energy, *T*: temperature, *S*: entropy.), in which the entropy for gas molecules was corrected based on experimental data^[^ ^45^ ^]^ and free energy correction for the adsorbates on active sites was achieved by computing the frequency of reaction intermediates with catalysts fixed. Detailed calculation information was described in support information.

## Conflict of Interest

The authors declare no conflict of interest.

## Supporting information



Supporting Information

## Data Availability

The data that support the findings of this study are available from the corresponding author upon reasonable request.;
