# Supplementary material for: Medium‐Entropy Alloy/Oxide Nano Composite for High‐Performing High‐Temperature CO2 Electrolysis with Remarkable Carbon Deposition Resistance
Source: Adv Sci (Weinh). 2025 Jul 29;12(39):e08800. doi: 10.1002/advs.202508800 (PMC12533207; doi:10.1002/advs.202508800)
Supplement: Supplementary file 1 — Supporting Information [file ADVS-12-e08800-s001.pdf]

ADVANCED SCIENCE
Open Access

Supporting Information

for *Adv. Sci.*, DOI 10.1002/advs.202508800

Medium-Entropy Alloy/Oxide Nano Composite for High-Performing High-Temperature CO_2 Electrolysis with Remarkable Carbon Deposition Resistance

Jun Tong, Haewon Seo, Yunseo Choi, Ji-eun Won, Jinhong Park, Keun Hwa Chae, Jongsup Hong, Hye Jung Chang, Baowen Zhou, Rongchang Cao, Na Ni, Kyung Joong Yoon*, Lei Zhu* and Zhen Huang*

Supporting information for
Medium-Entropy Alloy/Oxide Nano Composite for High-performing High-temperature CO_2 Electrolysis with Remarkable Carbon Deposition Resistance

Jun Tong[1,2], Haewon Seo[2], Yunseo Choi[3], Ji-eun Won[2,3], Jinhong Park[2,4], Keun Hwa Chae[5], Jongsup Hong[3], Hye Jung Chang[2], Baowen Zhou[1], Rongchang Cao[1], Na Ni[1], Kyung Joong Yoon[2]*, Lei Zhu[1]*, Zhen Huang[1]*

[1]Key Laboratory for Power Machinery and Engineering of Ministry of Education, Shanghai Jiao Tong University, Shanghai 200240, China

Email: tonyzhulei@sjtu.edu.cn, na.ni@sjtu.edu.cn

[2]Center for Hydrogen Energy Materials, Korea Institute of Science and Technology, Seoul 02792, Republic of Korea.

E-mail: kjyoon@kist.re.kr

[3]School of Mechanical Engineering, Yonsei University, Seoul 03722, Republic of Korea.

[4]Department of Chemical and Biological Engineering, Korea University, Seoul, 02841 Republic of Korea.

[5]Advanced Analysis Center, Korea Institute of Science and Technology, Seoul 02792, Republic of Korea.

Cell Preparation Detail: A multilayered ceramic structure, consisting of a support, fuel electrode functional layer, and electrolyte, was formed by sequence tape casting. Initially, 51 wt% NiO (FUJIFILM Wako Pure Chemical Corp., Japan), 34 wt% 3YSZ (TZ-3Y-E, Tosoh Corp., Japan), and 15 wt% poly (methyl methacrylate) (SUNPMMA-S50, Sunjin Beauty Science Co., Ltd., Korea) powders were ball-milled in ethanol with zirconia balls at 150 rpm for 24 h, with the addition of a dispersant (Triton X-100, Daejung Chemicals & Metals Co., Ltd., Korea). Subsequently, a binder (Butvar B-76, Eastman Chemical Company, USA) and plasticizers (polyethylene glycol 400 & glycerin, Daejung Chemicals & Metals Co., Ltd.) were added and mixed for another 24 h to obtain a homogeneous slurry.[1] Similarly, slurries were prepared for NiO (Sumitomo Metal Mining Co., Ltd., Japan)–8YSZ (TZ-8Y, Tosoh Corp.) (NiO/8YSZ = 66:34 wt%) fuel electrode functional layer and an 8YSZ electrolyte, using the same mixing procedure. To create fine pores, we added 1 wt% carbon black nanopowder (N550, OCI Company Ltd., Korea) as a pore former into the NiO–8YSZ slurry[1c, 2]. Each slurry was then degassed under 0.15 atm for 10 min during magnetic stirring in a vacuum desiccator, before being cast onto a glass substrate at a speed of 20 mm s^{-1}. After the layered tape had fully dried, it was cosintered at 1350 °C for 3 h and then cut into desirable sizes using a fiber laser cutter. Next, a GDC (GDC10-TC, Nexceris LLC, USA) interlayer was applied via spin coating at 6000 rpm for 60 s and sintered at 1250 °C for 2 h. Finally, an LSC (LSC64, Kceracell Co., Ltd., Korea) air electrode was screen-printed onto the surface of the GDC interlayer and then sintered at 950 °C for 2 h.

DFT Calculation Detail: In this study, a three-atomic-layer (111) facet of face-centered cubic nickel was constructed as the fundamental material to investigate metallic Ni and FCNC. Following that, the FCNC derived from pure Ni crystal was fabricated by high throughput screening method and the most stable one with lowest energy was sieved out for the catalytic computation.[3] The different metal atom sites in FCNC were considered as active site for solid-phase H_2 oxidation and CO_2 reduction. Similarly, the 111 surface of cubic MnO with two Mn–O layers was established as well. In order to demonstrate the synergistic effect between the FCNC and MnO components in the enhancement of catalytic performance, an in-plane binary heterojunction model of FCNC and MnO was rationally constructed. Specifically, a number of randomly-distributed FCNC part in the composite materials was firstly modelled, and a high throughput energy screening method was then employed to find out the most stable on with lowest energy which served as catalysts for reaction calculations. Notably, for the better exposure of the reactive sites at the heterojunction interface, the section of MnO protruding above the FCNC was removed while maintaining the stoichiometric ratio of Mn and O.

To adhere to the experimental reaction conditions, the free energy correction of all free gases and adsorbates was treated at 1 atm pressure and a temperature of 750 °C. Specially, there was an oxygen anion (O^{2-}) coupled with two electrons getting involved in the reaction pathway, leading to a grand challenge to directly calculate the free energy of both. As a result, similar to the computed hydrogen electrode ($H^+ + e^- = 1/2\ H_2$), the computed oxygen electrode ($O^{2-} + 2e^- = 1/2\ O_2$) was used to compute the free energy

of O^{2-} coupled with electrons.

Specially, in SOFC, the overall reaction of H_2 oxidation to H_2O can be expressed as:

H_2 (g) + O^{2-} (E) → H_2O + $2e^-$

In this process, H_2 reacts with oxygen anion in electrolyte to produce gaseous H_2O and two electrons transferred to electrode. Specifically, this whole reaction can be divided into four elementary steps (* denotes the active sites):

1. *+ H_2 → *H + *H
2. *H + *H + O^{2-} (E) → *H + *H + *O + $2e^-$
3. *H + *H +*O → *H + *OH
4. *H + *OH → * + H_2O

Another half reaction studied in this research is the reduction of CO_2 into CO, so the whole reaction equation can be given by:

CO_2 → CO + O^{2-} (E)

Where reactant CO_2 directly dissociates into CO and oxygen anion transferred into electrolyte, which consists of the following four elementary step:

1. * + CO_2 → *CO_2
2. *CO_2 → *CO +*O
3. *CO + *O → *CO + O^{2-} (E)
4. *CO → * + CO

For the calculation of CO cracking into carbon deposits, only the following single step was considered:

1. *CO → O^{2-} (E) + *C

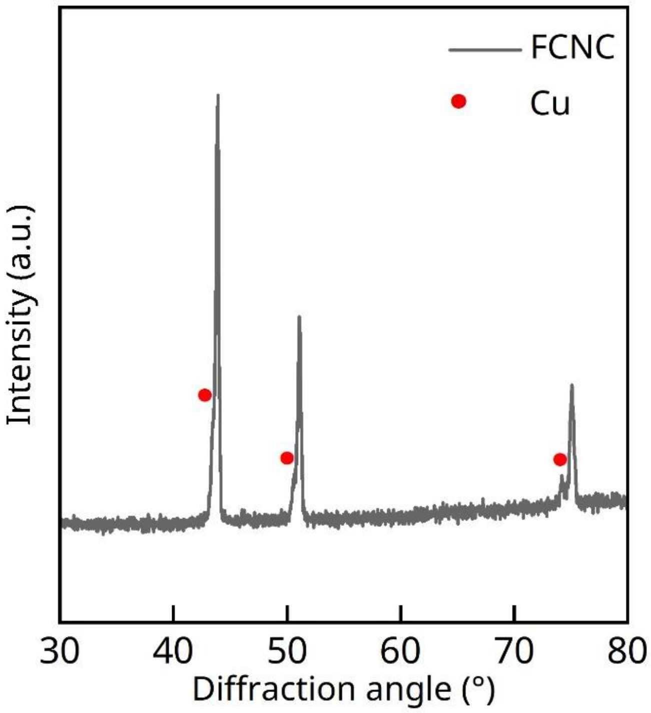

Figure S1. The XRD pattern of the FCNC alloy.

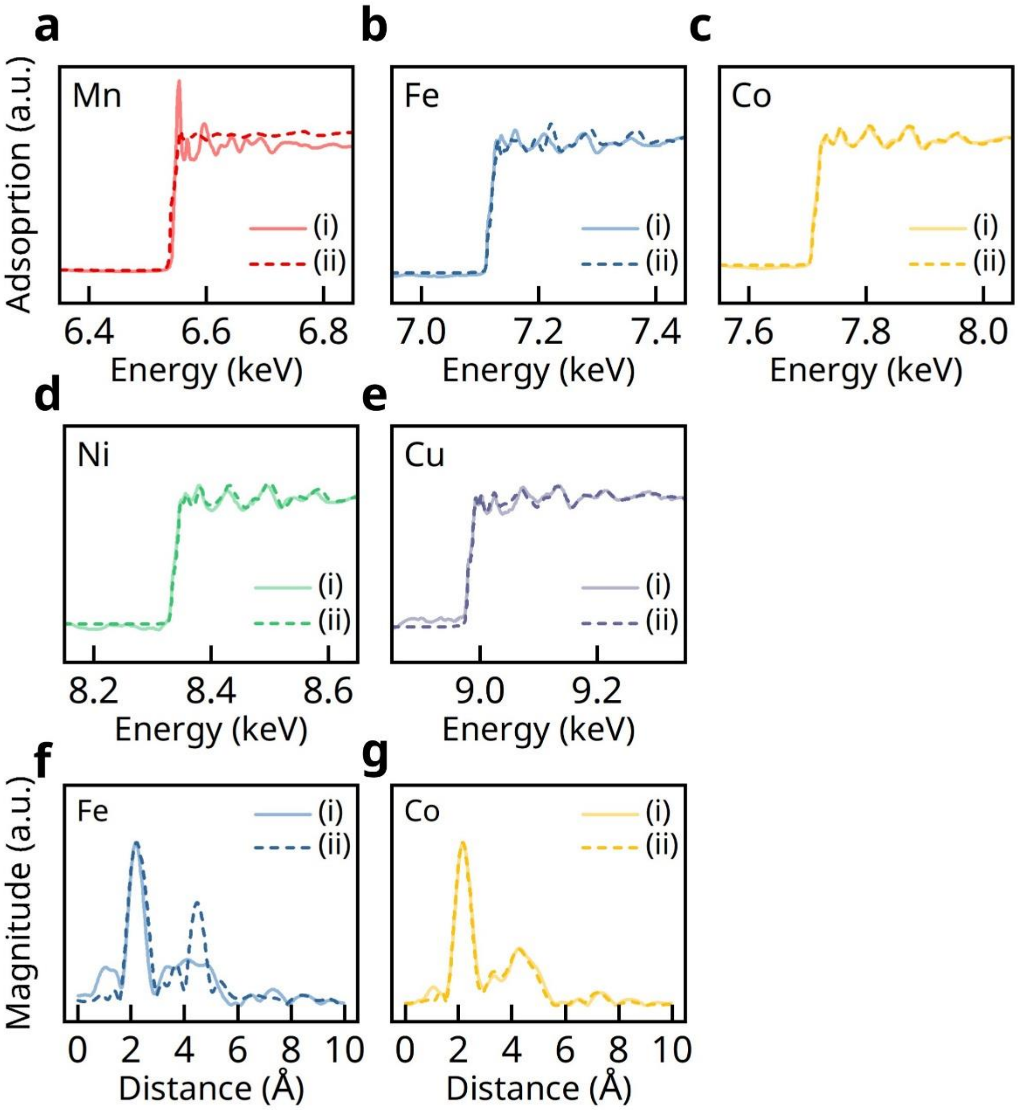

Figure S2. (a–e) XANES spectra for (a) Mn, (b) Fe, (c) Co, (d) Ni, and (e) Cu, along with (f–g) FT-EXAFS spectra for (f) Fe and (g) Co, were obtained from (i) FeCoNiCu/MnO after reduction and (ii) the corresponding foil reference materials.

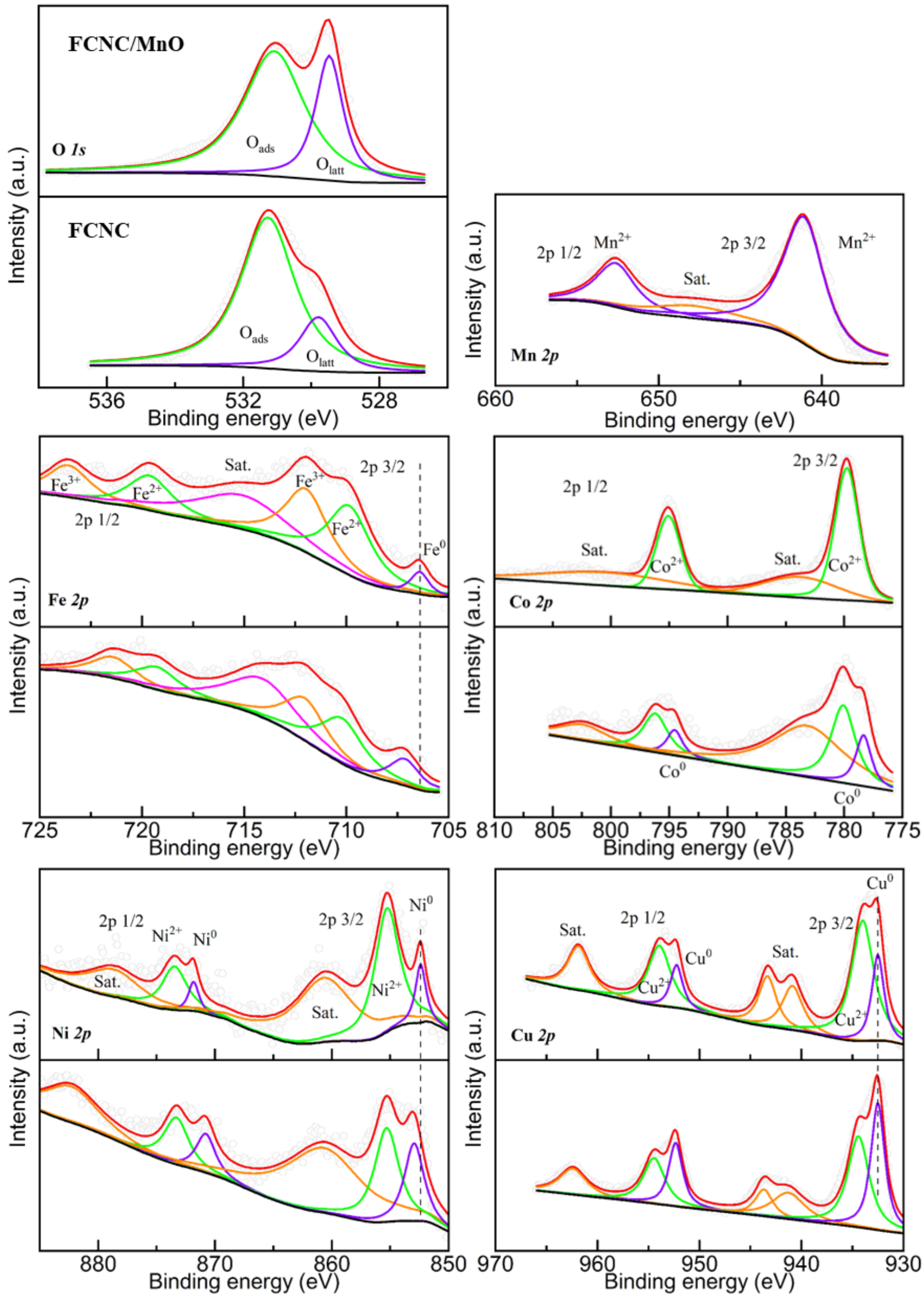

Figure S3. XPS spectra of the elements in FCNC and FCNC/MnO.

	Peak area percentage of the oxidation state signals in FCNC.	Peak area percentage of the oxidation state signals in FCNC/MnO.
Fe	82%	93%
Co	70%	100%
Ni	60%	83%
Cu	52%	72%
O_{latt}	20%	31%

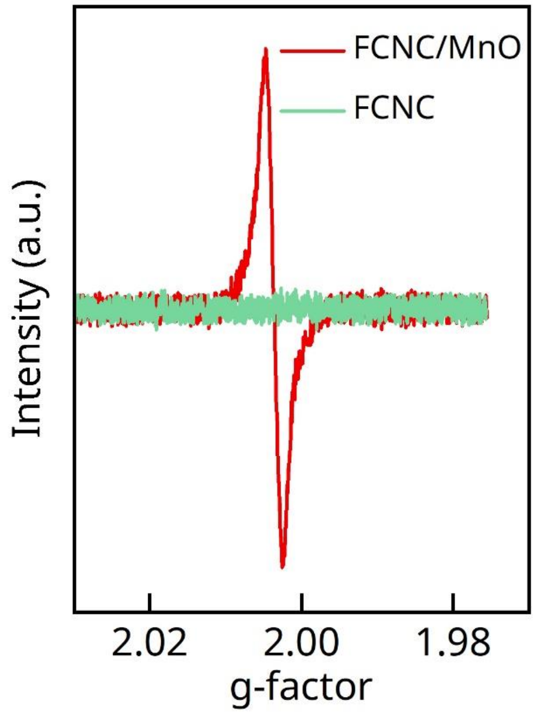

Figure S4. EPR spectra of FCNC/MnO and FCNC.

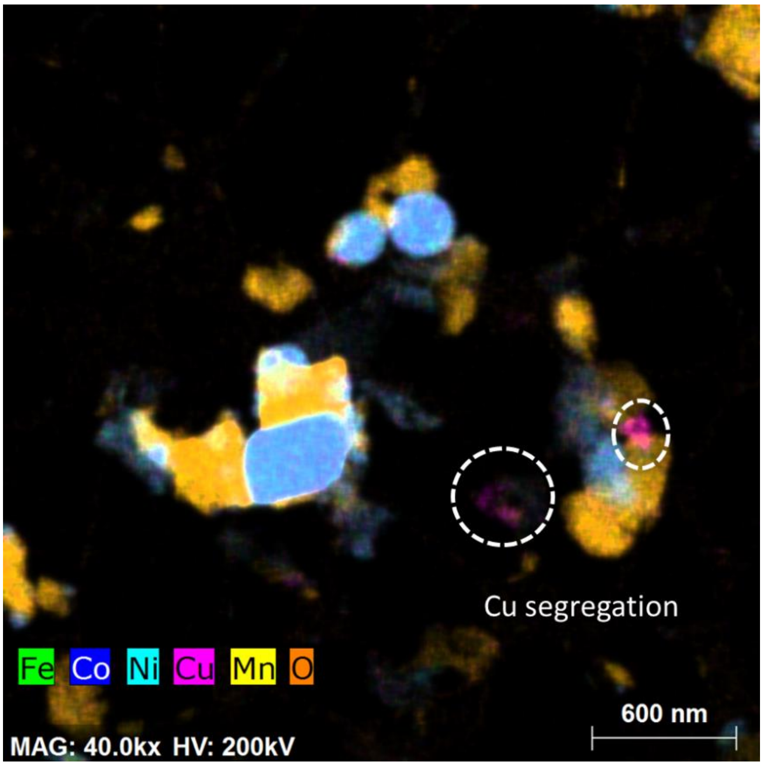

Figure S5. Low-magnification TEM image of FCNC/MnO.

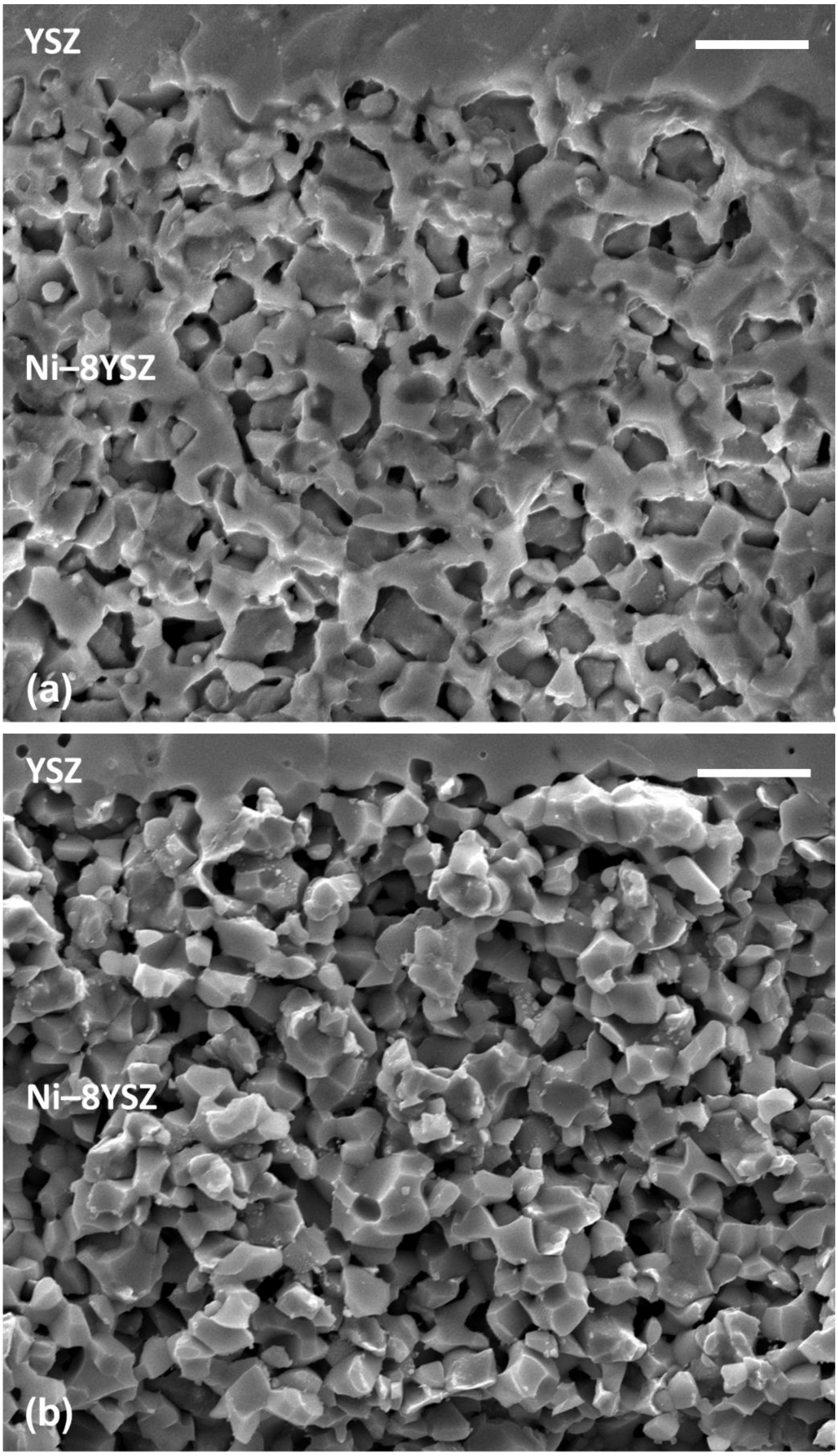

Figure S6. SEM images of the electrode (a) before and (b) after catalyst impregnation. The scale bar is 2μm.

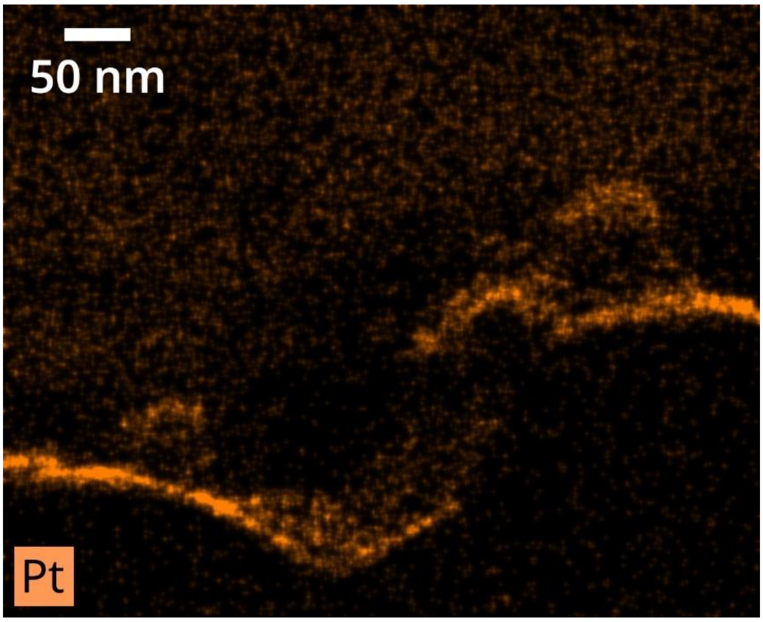

Figure S7. EDS map of Pt element.

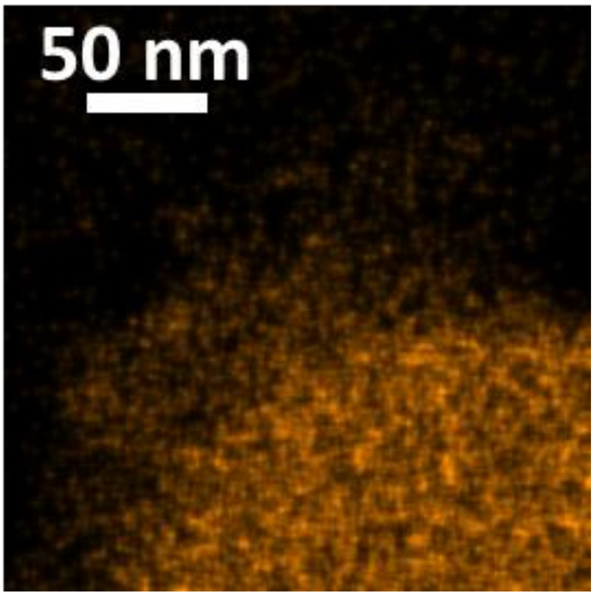

Figure S8. The EDS map of O element.

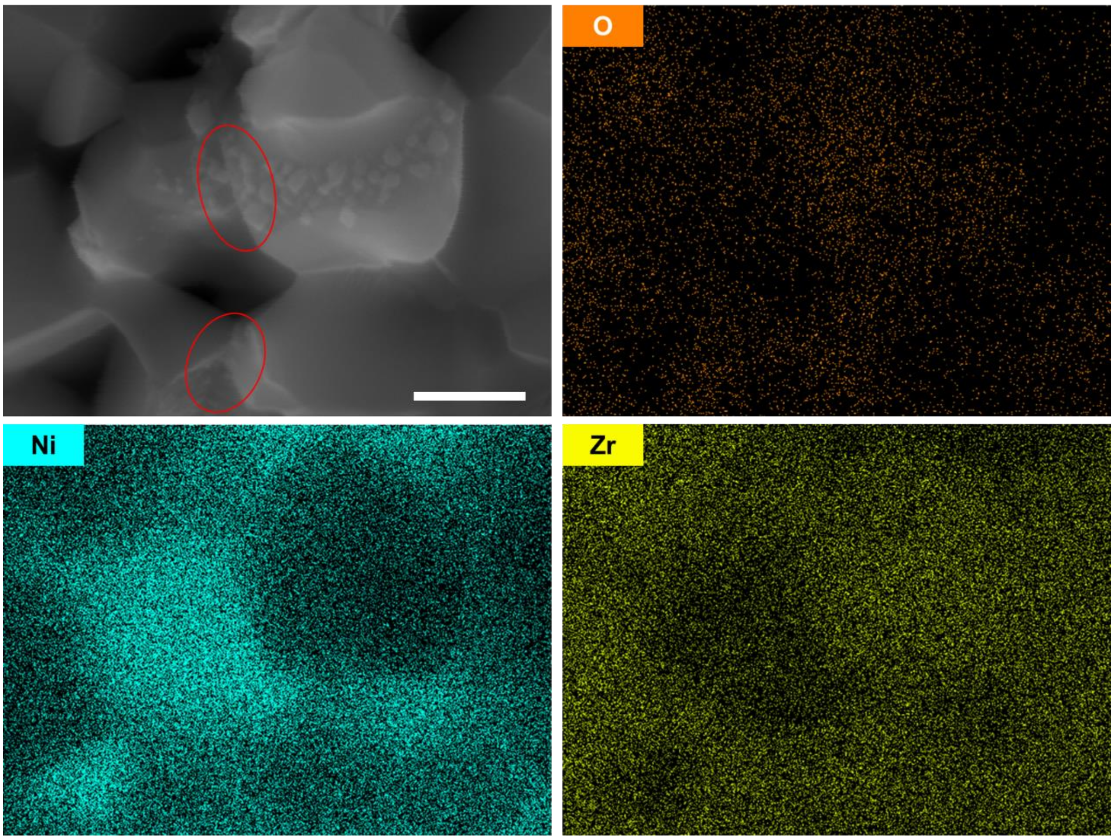

Figure S9. The SEM–EDS result of nanoparticles co-distributed on the Ni/8YSZ surface. The scale bar is 500 nm.
(Note: The EDS images were used only to assist in identifying the scaffold material, as the nanoparticles were too small to be detected by the EDS in the SEM equipment.)

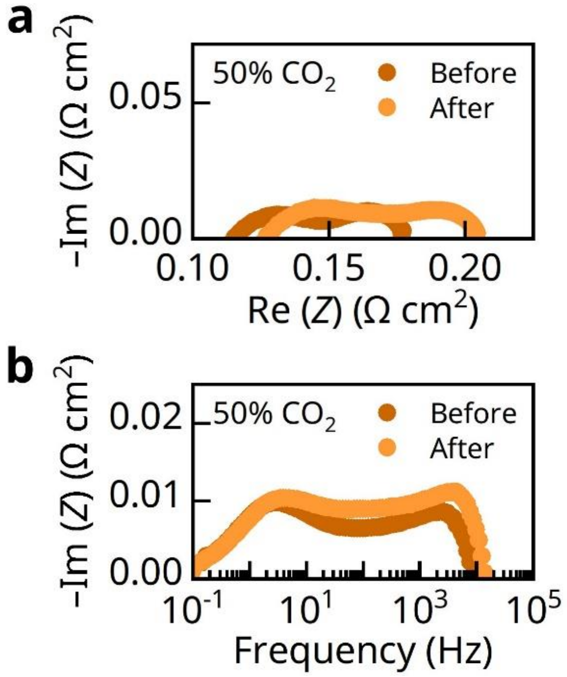

Figure S10. Nyquist and Bode plots of the impedance spectra for the FCNC/MnO cell before and after the anti-coking performance test.

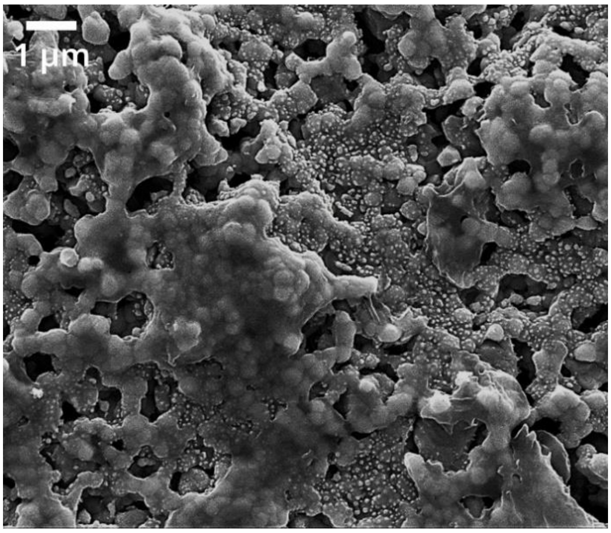

Figure S11. SEM image of the oxygen electrode surface after the durability test.

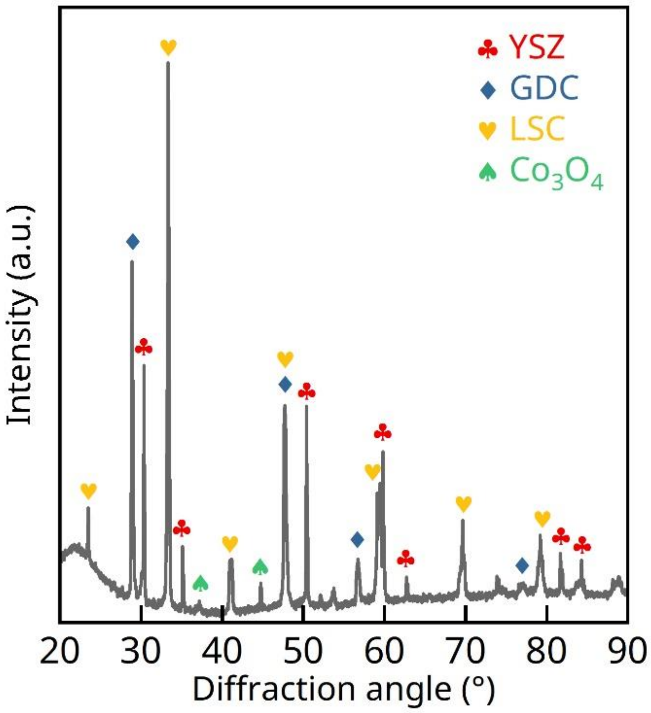

Figure S12. XRD pattern of the oxygen electrode surface after the durability test.

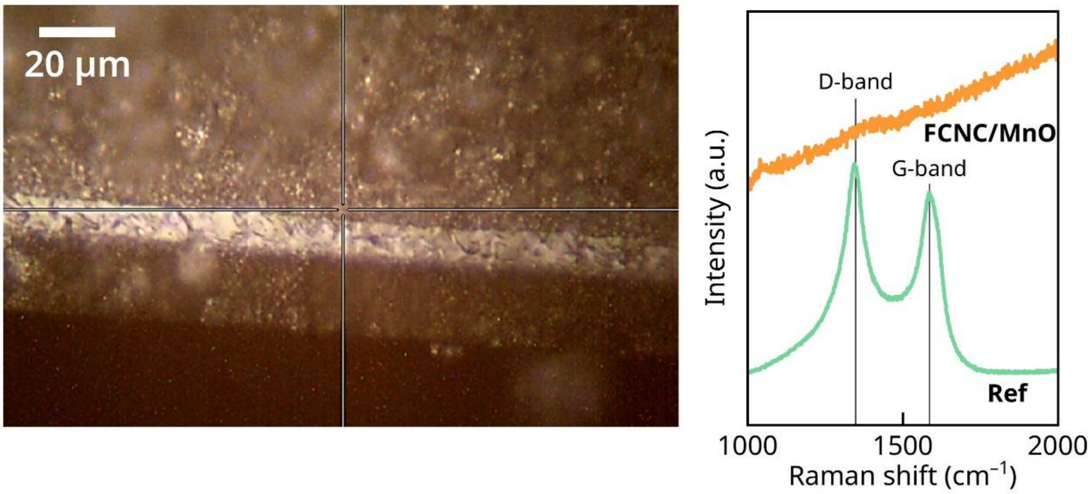

Figure S13. Raman spectra obtained from the Ni–8YSZ functional layer of the cells with and without FCNC/MnO infiltration. A scanning region photo is provided on the left, and the test results are displayed on the right.

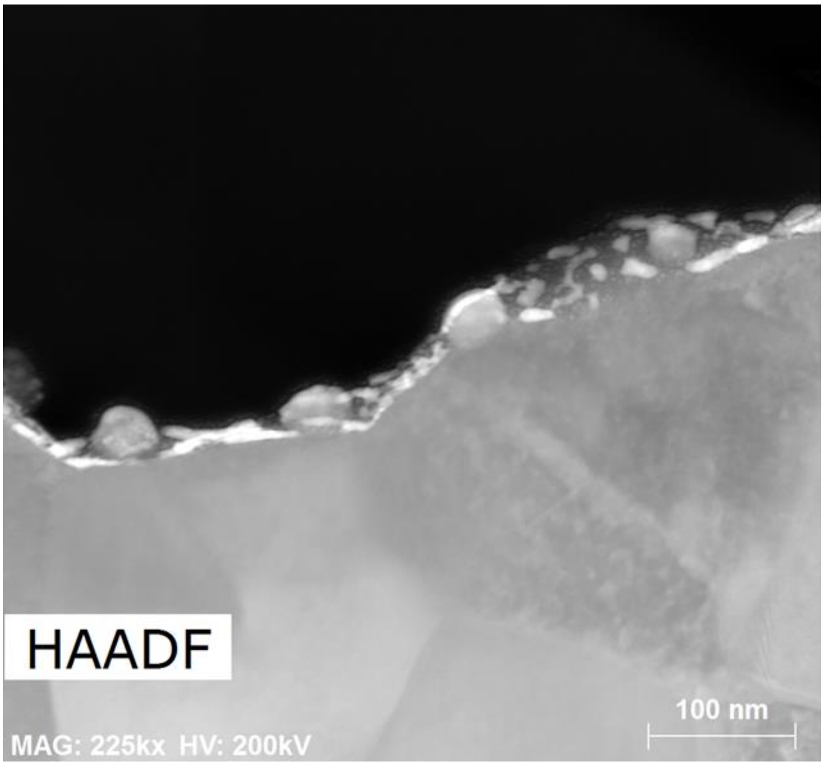

Figure S14. Displays the HAADF-STEM image of nanoparticles obtained from FIB-sectioning of the electrode of the FCNC/MnO-impregnated cell after the durability test.

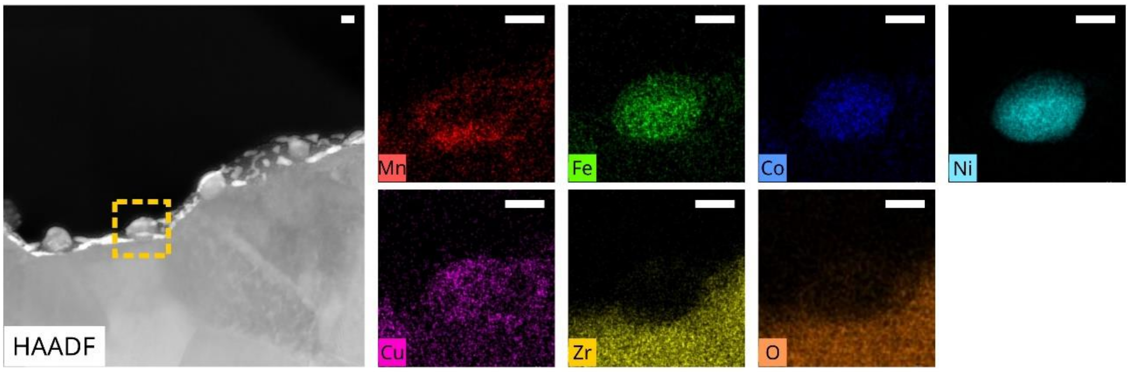

Figure S15. EDS results of the selected particle.

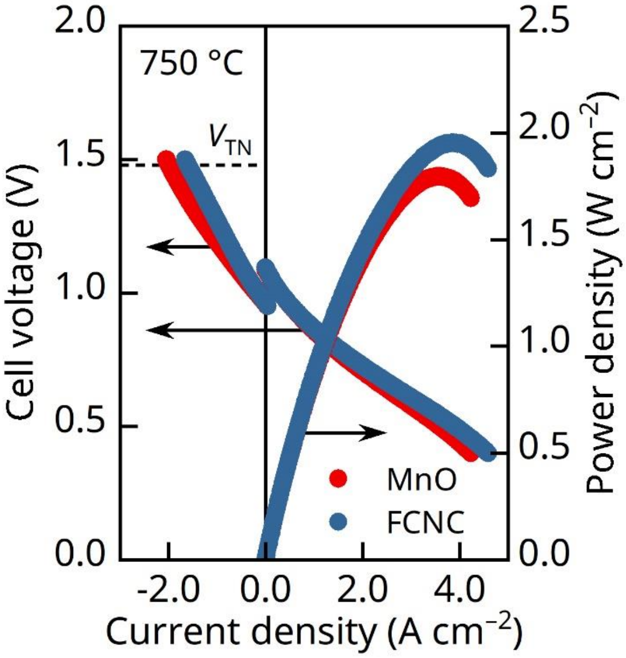

Figure S16. Current–voltage curves of the cells impregnated with FCNC and MnO in SOFC and SOEC modes at 750 °C.

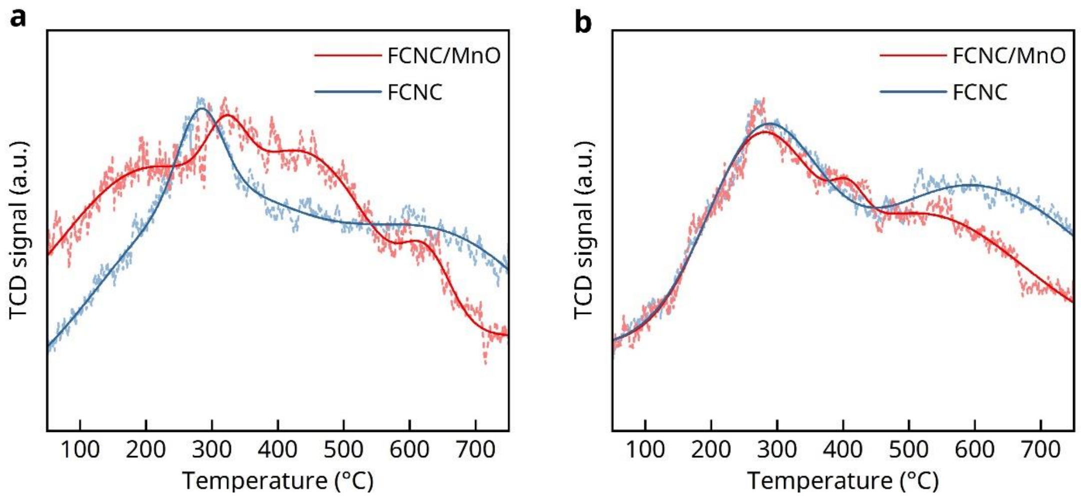

Figure S17. (a) H_2-TPD and (b) CO_2-TPD results of FCNC/MnO and FCNC.

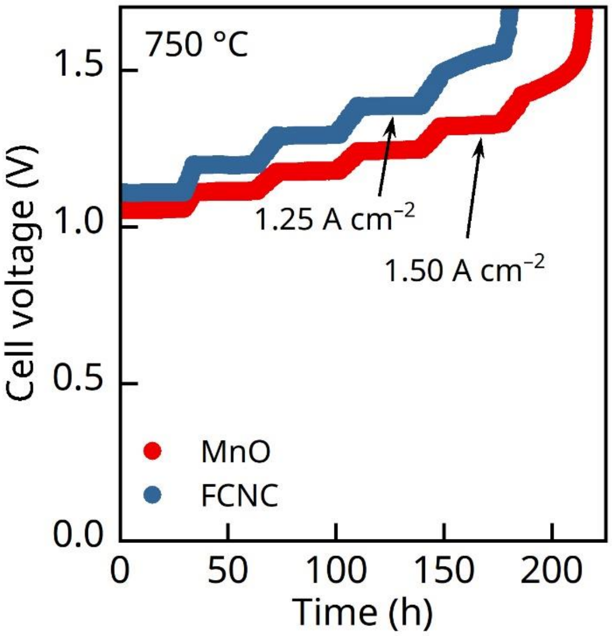

Figure S18. presents the anti-coking performance test results of the two cells in Figure S16.

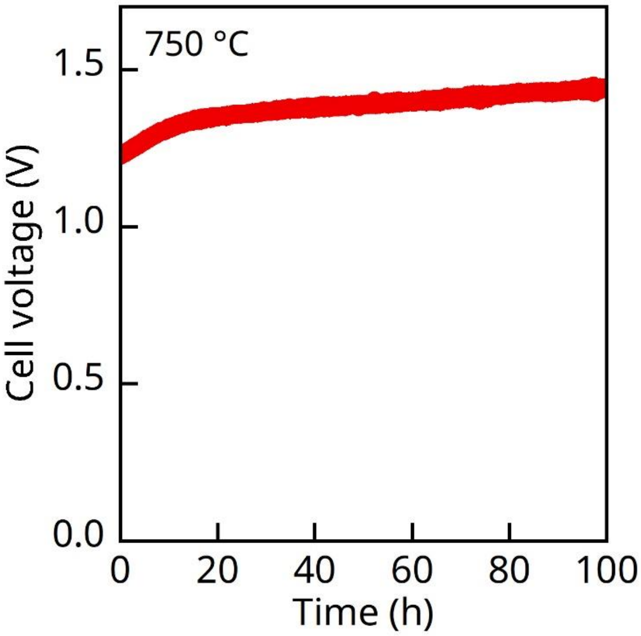

Figure S19. The durability test results of the MnO-impregnated cell at a current density of 1.0 A cm^{-2}.

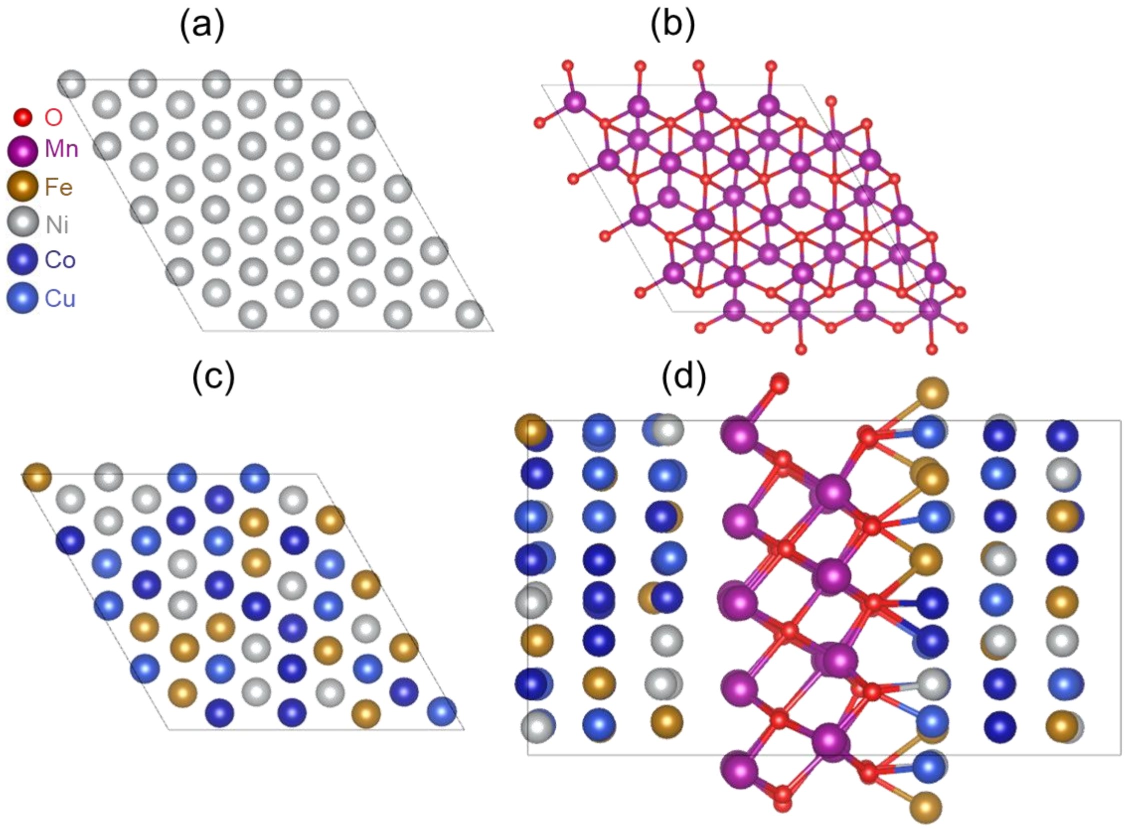

Figure S20. (a) Computational model of Ni; (b) computational model of MnO; (c) computational model of FCNC; (d) computational model of FCNC/MnO.

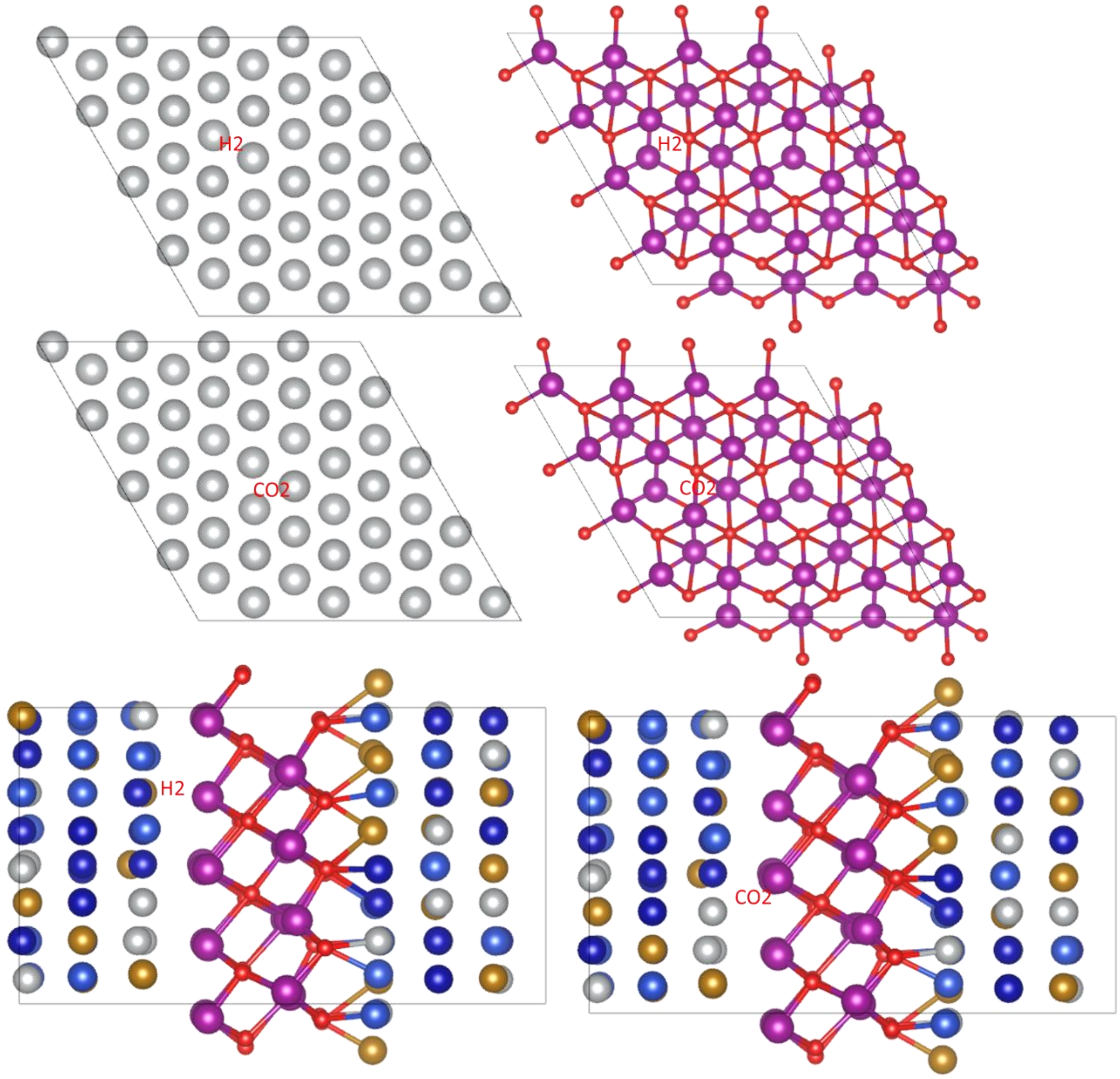

Figure S21. Computational binding sites of H_2 and CO_2 molecules on Ni, MnO and FCNC/MnO surfaces.

Table S1. Performance of Ni–YSZ fuel electrode-supported SOFCs with an LSC air electrode at 750 °C from other work.

Maximum power density ($W\ cm^{-2}$)	Reference
2.21	**This work**
1.5	Huang et al.[4]
1.1	Zheng et al.[5]
1.2	Fan et al.[6]
1.36	Rehman et al.[7]
1.75	Solovyev et al.[8]
1.2	Tong et al.[9]

Table S2. Performance of CO_2 electrolysis compared with other work.

Cell composition	Temp (°C)	Current density (A cm^{-2}) @1.5 V	Reference
FCNC/MnO@Ni/YSZ-YSZ-LSC	750	2.15	**This work**
$SF_{1.5}MC$-LSGM-PBSCF	750	~1.5	He et al.[10]
PSNFM-NFA@FeO-LSGM-LSCF	800	<2.0	Tan et al.[11]
SFRuM-LSGM-BSCF	800	<1.2	Lv et al.[12]
Ru_1/SDC-LSGM-LSCF	800	~2.1	Song et al.[13]
430 stainless steel-YSZ-LSCF	750	0.71	Zhang et al.[14]
LSCFN-YSZ-LSCFN	850	<0.7	Yang et al.[15]
Ni/YSZ-YSZ-LSM	800	<1.0	Wang et al.[16]
Ni/YSZ-YSZ-LSCF	800	<0.6	Wang et al.[17]
SFMNi-F -LSGM- SFMNi-F	750	~1.8	Zhang et al.[18]

Reference

[1] a)H. Seo, M. Kishimoto, C. Ding, et al., Improvement in the Electrochemical Performance of Anode-supported Solid Oxide Fuel Cells by Meso- and Nanoscale Structural Modifications, *Fuel Cells* **2020**, 20, 570. https://doi.org/10.1002/fuce.202000079; b)C. Ding, H. Seo, M. Kishimoto, H. Iwai, Temperature-controlled microextrusion printing for mesoscale interfacial designing in solid oxide fuel cells, *Fuel Cells* **2023**, 23, 264. https://doi.org/10.1002/fuce.202200170; c)J. Min, H. Seo, J. Shin, et al., Atomically dispersed platinum electrocatalysts supported on gadolinia-doped ceria nanoparticles for practical high-temperature solid oxide cells, *Journal of Materials Chemistry A* **2023**, 11, 25298. 10.1039/D3TA05534E

[2] H. Seo, S. Jang, W. Lee, et al., Highly efficient, coke-free electrolysis of dry CO2 in solid oxide electrolysis cells, *Chemical Engineering Journal* **2024**, 481, 148532. https://doi.org/10.1016/j.cej.2024.148532

[3] Y. Shao, J. Ni, J. Yin, et al., Fe-Rich Medium-Entropy Core-Shell Electrocatalyst for Hydrogen Evolution Reaction Under Large Current Density, *Small* **2025**, 21, 2407061. https://doi.org/10.1002/smll.202407061

[4] Z. Huang, Z. Liu, H. Hu, et al., Evaluation of La0.6Sr0.4CoO3-δ-Ce0.85Sm0.075Nd0.075O2-δ composite cathodes for intermediate temperature solid oxide fuel cells, *Ceramics International* **2022**, 48, 16319. https://doi.org/10.1016/j.ceramint.2022.02.182

[5] Z. Zheng, J. Jing, Z. Yang, et al., Stability evaluation and quantitative analysis of filmy cathode in solid oxide fuel cells under operating conditions, *International Journal of Hydrogen Energy* **2023**, 48, 4446. https://doi.org/10.1016/j.ijhydene.2022.10.273

[6] H. Fan, Z. Liu, Y. Wu, et al., Electrochemical performance of La0.6Sr0.4CoO3—Ce0.9Gd0.1O1.95 composite cathode for IT-SOFCs, *International Journal of Applied Ceramic Technology* **2024**, 21, 289. https://doi.org/10.1111/ijac.14490

[7] S. U. Rehman, M. H. Hassan, H.-S. Kim, et al., Designing the nano-scale architecture of the air electrode for high-performance and robust reversible solid oxide cells, *Applied Catalysis B: Environment and Energy* **2023**, 333, 122784. https://doi.org/10.1016/j.apcatb.2023.122784

[8] A. A. Solovyev, K. A. Kuterbekov, S. A. Nurkenov, et al., Anode-supported solid oxide fuel cells with multilayer LSC/CGO/LSC cathode, *Fuel Cells* **2021**, 21, 408. https://doi.org/10.1002/fuce.202000168

[9] X. Tong, C. Li, K. Xu, et al., Nanoengineering of electrodes via infiltration: an opportunity for developing large-area solid oxide fuel cells with high power density, *Nanoscale* **2023**, 15, 16362. 10.1039/D3NR02704J

[10] F. He, M. Hou, F. Zhu, et al., Building Efficient and Durable Hetero-Interfaces on a Perovskite-Based Electrode for Electrochemical CO2 Reduction, *Advanced Energy Materials* **2022**, 12, 2202175. https://doi.org/10.1002/aenm.202202175

[11] T. Tan, Z. Wang, M. Qin, et al., In Situ Exsolution of Core-Shell Structured NiFe/FeOx Nanoparticles on Pr0.4Sr1.6(NiFe)1.5Mo0.5O6-δ for CO2 Electrolysis, *Advanced Functional Materials* **2022**, 32, 2202878. https://doi.org/10.1002/adfm.202202878

[12] H. Lv, L. Lin, X. Zhang, et al., Promoting exsolution of RuFe alloy nanoparticles on Sr2Fe1.4Ru0.1Mo0.5O6−δ via repeated redox manipulations for CO2 electrolysis, *Nature Communications* **2021**, 12, 5665. 10.1038/s41467-021-26001-8

[13] Y. Song, J. Min, Y. Guo, et al., Surface Activation by Single Ru Atoms for Enhanced High-Temperature CO2 Electrolysis, *Angewandte Chemie International Edition* **2024**, 63, e202313361. https://doi.org/10.1002/anie.202313361

[14] B. Zhang, S. Zhang, Z. Zhang, K. Tang, C. Xia, Metal-supported solid oxide electrolysis cell for direct CO2 electrolysis using stainless steel based cathode, *Journal of Power Sources* **2023**, 556, 232467. https://doi.org/10.1016/j.jpowsour.2022.232467

[15] Z. Yang, C. Ma, N. Wang, et al., Electrochemical reduction of CO2 in a symmetrical solid oxide electrolysis cell with La0.4Sr0.6Co0.2Fe0.7Nb0.1O3-δ electrode, *Journal of CO2 Utilization* **2019**, 33, 445. https://doi.org/10.1016/j.jcou.2019.07.021

[16] H. Wang, F. Jiang, X. Wang, L. Ye, K. Xie, Enhanced CO2 Electrolysis with the Modification of Ni–YSZ Cathodes by SDC in Microtubular Solid Oxide Electrolysis Cells, *Energy & Fuels* **2022**, 36, 13195. 10.1021/acs.energyfuels.2c02788

[17] T. Wang, J. Wang, L. Yu, et al., Effect of NiO/YSZ cathode support pore structure on CO2 electrolysis via solid oxide electrolysis cells, *Journal of the European Ceramic Society* **2018**, 38, 5051. https://doi.org/10.1016/j.jeurceramsoc.2018.07.005

[18] S. Zhang, Y. Jiang, H. Han, Y. Li, C. Xia, Perovskite Oxyfluoride Ceramic with In Situ Exsolved Ni–Fe Nanoparticles for Direct CO2 Electrolysis in Solid Oxide Electrolysis Cells, *ACS Applied Materials & Interfaces* **2022**, 14, 28854. 10.1021/acsami.2c05324
